# The Chinese Medicine Wu-Tou Decoction Relieves Neuropathic Pain by Inhibiting Hippocampal Microglia Activation

**DOI:** 10.1038/s41598-018-30006-7

**Published:** 2018-08-16

**Authors:** Chunyan Zhu, Qionghong Xu, Zhiyun Mao, Na Lin

**Affiliations:** Institute of Chinese Materia Medica, China Academy of Chinese Medical Sciences, Beijing, 100700 China

## Abstract

The comorbidity between the nociceptive and mental syndromes adds to the refractoriness of neuropathic pain (NP). Wu-Tou decoction (WTD) has been prescribed for chronic pain for thousands of years in China. Recently, we reported that WTD was helpful for hippocampus and co-curative for the nociceptive, depressive and anxiety behaviors in the spinal cord ligation (SNL) mice. However, the mechanism underlying the rescue of hippocampus, as well as the roles hippocampus assumed in co-curation remain unexplored. In this study, we validated that in SNL mice, the long-lasting damages to limbic system were mainly limited to hippocampus. In addition, hippocampal neurons were proven sensitive to harms induced by microglia and rescued by WTD, which in sum indicated hippocampal microglia as the critical modulator of co-curation. To validate this hypothesis the hippocampal microglia were mal-activated in shamed mice, in which the atrophy of hippocampus and the development of NP syndromes were consolidated and proven rescued by WTD. On the contrary, in the SNL mice, the failure to control hippocampal microglia was sufficient to void all the rescues mediated by WTD. In sum, our study points out that the effective modulation of microglia in hippocampus is of pivotal importance for the co-curation by WTD.

## Introduction

Pain is a kind of complex sensory and mental experience, including not only the actual damage to the tissue but also the comprehension, description and the cognition of the injures. Especially, for the chronic stage of pain, in which the initial damage has long been recovered, the perception of pain has been proven more dependent on the mental state rather than the actual damages^1^.

Neuropathic pain (NP), triggered by various kinds of the lesions to the somatosensory system, is a kind of refractory chronic pain characterized by high clinical relevance. As high as 61.9% and 54.3% of NP patients have been beset by the serious depression and anxiety respectively^2^. In addition, the damages to mental health have been proven aggravated by the severity of pain. The vicious cycle between the nociceptive and mental deficits adds to the severity and refractoriness of NP. Patients with mental syndromes are proven not only more sensitive to pain, but also more insensitive to the first-line analgesic drugs^2–4^. Therefore, the effective control of mental syndromes is urgent in clinical treatments.

Multiple studies focusing on the mechanism of comorbidity propose that chronic pain is a maladaptive neuropathological disease state, in which the alternations of the activities and circuitries between brain nuclei in sum contribute to the addition to pain^5^.

Especially, alternations associated with hippocampus have been widely discussed, associated with the co-curation in both the clinical patients and animal models of NP^6–10^. However, the causal link between hippocampus and the comorbidity, as well as the mechanism underlying the functional alternations in hippocampus remain unexplored, which greatly limited the discovery of drugs focusing on the co-curation.

In this study, we focus on microglia as the modulator of the functional alternations in hippocampus. There is compelling evidence that the microglia in brain not only negatively monitor the synaptic function, but also actively regulate the plasticity of neurons^11^. The morphological retraction of the ramified processes, the biochemical secretion of TNFα, as well as the metabolism of the neurotransmitters and the receptors in sum contribute to the microglia mediated remodeling of the central nerves system, especially in conditions of chronic pain^12^.

Especially, the up-regulation of tumor necrosis factor alpha (TNFα), secreted by the activated microglia in hippocampus, have been widely recognized as the linker between the comorbidity of chronic pain and the emotional syndromes^13^. Firstly, the mal-regulation of TNFα in central nerves system is prevalent in patients and animal models^14^ suffering from NP^15^. Secondly, the pre-clinic studies show that the up-regulated TNFα in brain is related with the development of nociceptive^16^ and cognitive syndromes^17^ in naïve ones, as well as the occurrence of the depressive^18^ and cognitive syndromes^17^ in NP ones. In addition, the TNFR1 receptor has been proven essential for the development of depressive syndromes in NP animals^18^. Lastly, both the down-regulation of TNFα by antibodies and the inhibition of TNFα receptors by antagonist were reckoned effective in the remission of pain and depression^19,20^. The commercialized drugs such as infliximab and etanercept have been clinically applied in arthritis and proven effective for the depressive symptoms for a subtype of patients characterized by the elevated expression of inflammatory factors in the blood^21,22^.

Wu-Tou decoction (WTD) has been characterized by the anti-inflammatory functions and clinically applied for chronic pain for thousands of years in China. The prescription of WTD is composed of Radix Aconiti, Herba, Ephedrae, Radix Astragali, Raidix Paeoniae Alba and Radix Glycytthizae. In the water extraction, 74 components assuming the anti-inflammation and oxidant functions have been identified and reckoned as quality control^23,24^. In previous studies, we illustrated that the anti-inflammation effects of WTD are not limited in the peripherical nerves system. In both spinal cord^25,26^ and hippocampus^27^, the effective controlling of pro-inflammatory cytokines has been consolidated in NP models. However, the causal link between the anti-inflammation in hippocampus and co-curation by WTD remain unexplored.

To verify the brain nuclei responsible for the comorbidity and curation, we traced the morphological changes of neurons in anterior cingulate cortex (ACC), the basolateral amygdala (BLA), hippocampal CA1 and CA3 in the whole pathological progress. In ACC and BLA, the atrophies were found in the early stage, but validated self-healing in the late stage when the mental deficits were still lasting. Only in hippocampus, the progressive atrophy was consolidated long-lasting and accompanied by the over-activation of microglia in SNL, which were totally relieved by WTD. On this basis, we hypothesized that: 1. Hippocampus is one of the most important brain nuclei mediating the maintenance of NP syndromes and the co-curation by WTD; 2. The over-expressed TNFα in hippocampus contributes to the over-activation of microglia, which further leads to the atrophy of hippocampus and the development of NP syndromes; 3. The modulation of the hippocampal microglia is responsible for the pain, depression and anxiety co-curation by WTD. For this aim, the hippocampal microglia were over-activated in naïve mice to evaluate the following NP syndromes, as well as the anti-inflammation, neuronal protection and the co-curative effects of WTD. Further, to clarify whether the modulation of hippocampal microglia is pivotal for co-curation, the WTD-mediated down-regulation of hippocampal TNFα was offset in SNL mice. The following consequences of the offset were analyzed by the bio-chemical, morphological and behavioral detections. In addition, to evaluate the harms microglia made to the hippocampus and the rescue by WTD in more direct way, the double staining of the glutamatergic and GABAergic neurons was performed in hippocampus of the sham, SNL with/without WTD, hippocampal TNFα injection with/without WTD, and the SNL-WTD with hippocampal injection of TNFα mice.

## Results

### The atrophy of limbic system in SNL mice was found long-lasting in hippocampus and rescued by WTD

To clarify the brain nuclei, especially nuclei of the limbic system, responsible for the comorbidity and co-curation in NP, the morphological atrophy and remission in ACC, BLA, CA1 and CA3 were analyzed by Golgi staining (detailed data were shown in supplementary sheet 1.1–1.5).

For ACC (Supplemental Fig. 1A), the atrophy in SNL was limited in early stage (D3–10) as compared to Sham; For BLA (Supplemental Fig. 1B), the self-healing was also consolidated as early as D18. In sum, the self-healing in SNL indicated that ACC and BLA were not responsible for the long-lasting comorbidity.

**Figure 1:**
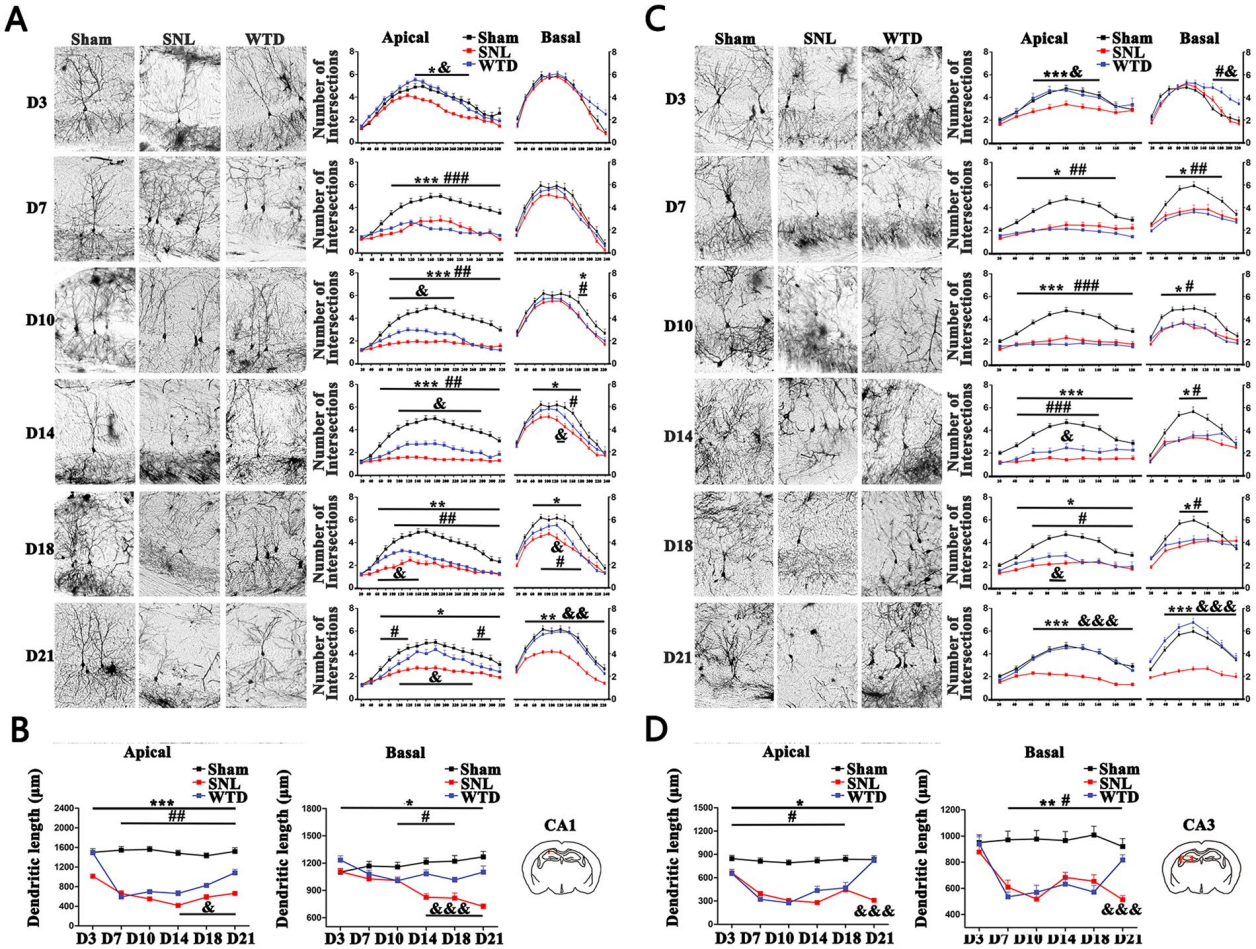
The SNL induced morphological alternations in hippocampus and the remission by WTD. (**A**,**B**) and (**C**,**D**) present the morphological alternations on the right side of CA1 and CA3 (n = 4 mice/group). In (**A**) and (**C**), the figures on the left side show the imaging of hippocampal pyramidal neurons detected on D3/7/10/14/18/21 in Sham/SNL/WTD groups, scale bar 100 μm; the scatter diagrams on the right show the statistical data of the intersections of dendrites on both the apical and basal sides. In (**B**) and (**D**), figures show the analyzations of the total length of dendrites on both the apical and basal sides. (*P < 0.05, **P < 0.01, ***P < 0.001 present the significant differences between the Sham and SNL groups; ^#^P < 0.05, ^##^P < 0.01, ^###^P < 0.001 present the significant differences between the Sham and WTD groups; ^&^P < 0.05, ^&&^P < 0.01, ^&&&^P < 0.001 present the significant differences between the SNL and WTD groups) (Data are shown as Mean ± SEM).

On the contrary, for CA1 (Fig. 1A) and CA3 (Fig. 1B), the sustained atrophy, represented by the decreases of the total length and intersections during the whole pathological process as compared to Sham, were consolidated permanent in SNL, indicating the important roles hippocampus assumed in the comorbidity.

In addition, the permanent damages in CA1 and CA3 were relieved by WTD. For CA1, the WTD mediated remission, shown by the increases of the intersection (Fig. 1A) and length (Fig. 1B) on both apical and basal sides as compared to SNL, were consolidated. However, the remission, shown by the significant differences between WTD and Sham in both the intersection and length on apical side, was far from recovery. For CA3, excepted for the transient and minor increases of intersections on D3/14/18, no significant increases were achieved by WTD prior to D21, as compared to SNL. However, on D21, significant increases of intersection (Fig. 1C) and length (Fig. 1D) were achieved by WTD on both apical and basal sides as compared to SNL. No significant differences were found between WTD and Sham on D21, indicating CA3 as the main target for WTD.

In sum, the persist brain damages in NP, which may be responsible for the sustained nociceptive and mental syndromes, were mainly restricted to hippocampus. The co-curative effects of WTD, as well as the rescue in CA3, indicate the important roles CA3 assumed in the comorbidity and co-curation.

### The hippocampal microglia may be responsible for the damages to hippocampal neurons, which were rescued by WTD

Given the permanent damages to hippocampus, as well as the mal-expression of TNFα in hippocampus reported in both animal models and patients suffering from NP, we further explored the roles hippocampal microglia assumed in damages in hippocampus and the rescue.

**Figure 2:**
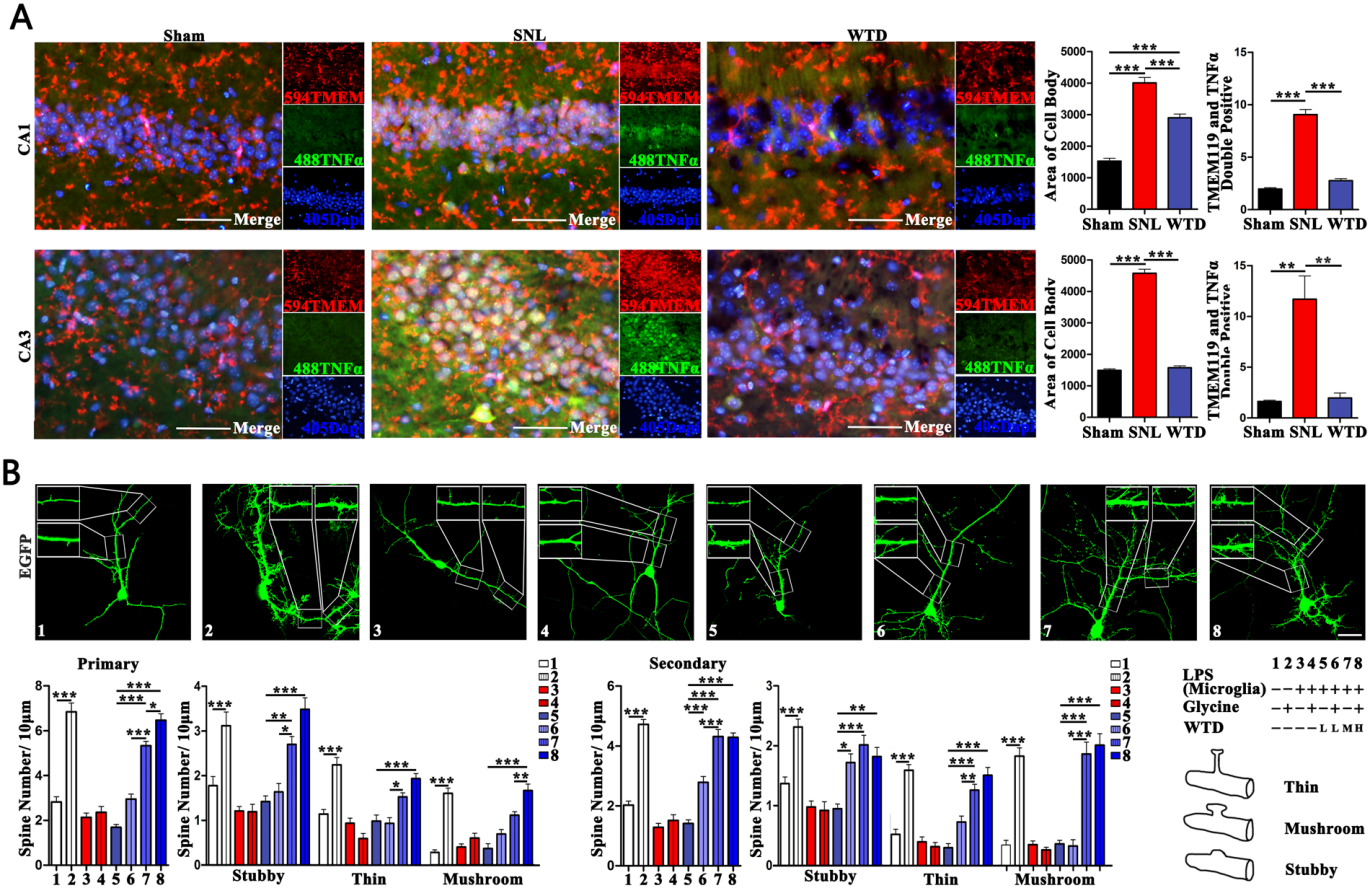
WTD inhibited the over-activated hippocampal microglia in SNL mice and rescued the damages over-activated microglia made to the cultured hippocampal neurons. (**A**) includes figures showing the morphological alternations of hippocampal microglia (scale bar 50 μm, right brain) on the left, the quantification of the area of cell body and the number of cells double stained by TMEM119 and TNFα on the right (*P < 0.05, **P < 0.01, ***P < 0.001, Mean ± SEM, n = 3 mice/group). (**B**) Shows the Z-projection confocal images of the GFP labelled neurons in top (scale bar 10 μm), the bar figures (*P < 0.05, **P < 0.01, ***P < 0.001, Mean ± SEM, at least 30 neurons pictured from 3 independent experiments are analyzed in each group) and the grouping information in bottom.

For this aim, the double staining of the microglia marker TMEM119 and TNFα were conducted to analyze the activation of hippocampal microglia (Fig. 2A). Compared to Sham, the SNL operations compromised the amplification of body size as well as the increase of double-stained cells in CA1 and CA3. In addition, compared to the SNL, the administration of WTD significantly relieved the amplification of cell bodies and the over-expression of TNFα in microglia marked by TMEM119 in CA1 and CA3. Although significant differences of cell body in CA1 still existed between WTD and Sham (detailed data were shown in supplementary sheet.2.1).

Further, by the quantitative analysis of the total length/intersections of dendrite and number/shape of dendritic spine, we validated whether the over-activated microglia are harmful to the plasticity of hippcampal neurons and the protections by WTD *in vitro* (Fig. 2B). For neurons incubated with the medium of the resting microglia (Group1 and 2), the chemical activation, represented by the increases of stubby/thin/mushroom spines in both the primary and secondary dendrites, were observed when exposed to glycine. On the contrary, for neurons incubated with the medium of the microglia activated by lipopolysaccharide (LPS)(Group 3 and 4), no significant increases were induced by glycine, which suggest that the over-activation of microglia is harmful to the plasticity of neurons. Furthermore, for neurons cultured in the medium of microglia activated by LPS and pretreated with WTD, the glycine mediated increases of spine density were achieved by WTD at the low (Group 6), medium (Group 7) and high (Group 8) doses as compared to Group 4. In sum, WTD was proven not only sufficient to inhibit the SNL induced over-activation of hippocampal microglia, but also protective for hippocampal neurons harmed by over-activated microglia *in vitro* (detailed data were shown in supplementary sheet.2.2).

### The mal-activation of hippocampal microglia was inducive of the damages in hippocampus and the comorbidity of NP syndromes, which were rescued by WTD

Given the important roles microglia assumed in the modulation of hippocampal neurons *in vitro*, we further explored whether there exists the causal link between the hippocampal microglia and the co-morbidity of NP syndromes, as well as the curation by WTD *in vivo*. To achieve these goals, the purified TNFα was injected into the CA3 of mice for the consecutive 7days. The following consequences of injection and the administration of WTD were analyzed.

**Figure 3:**
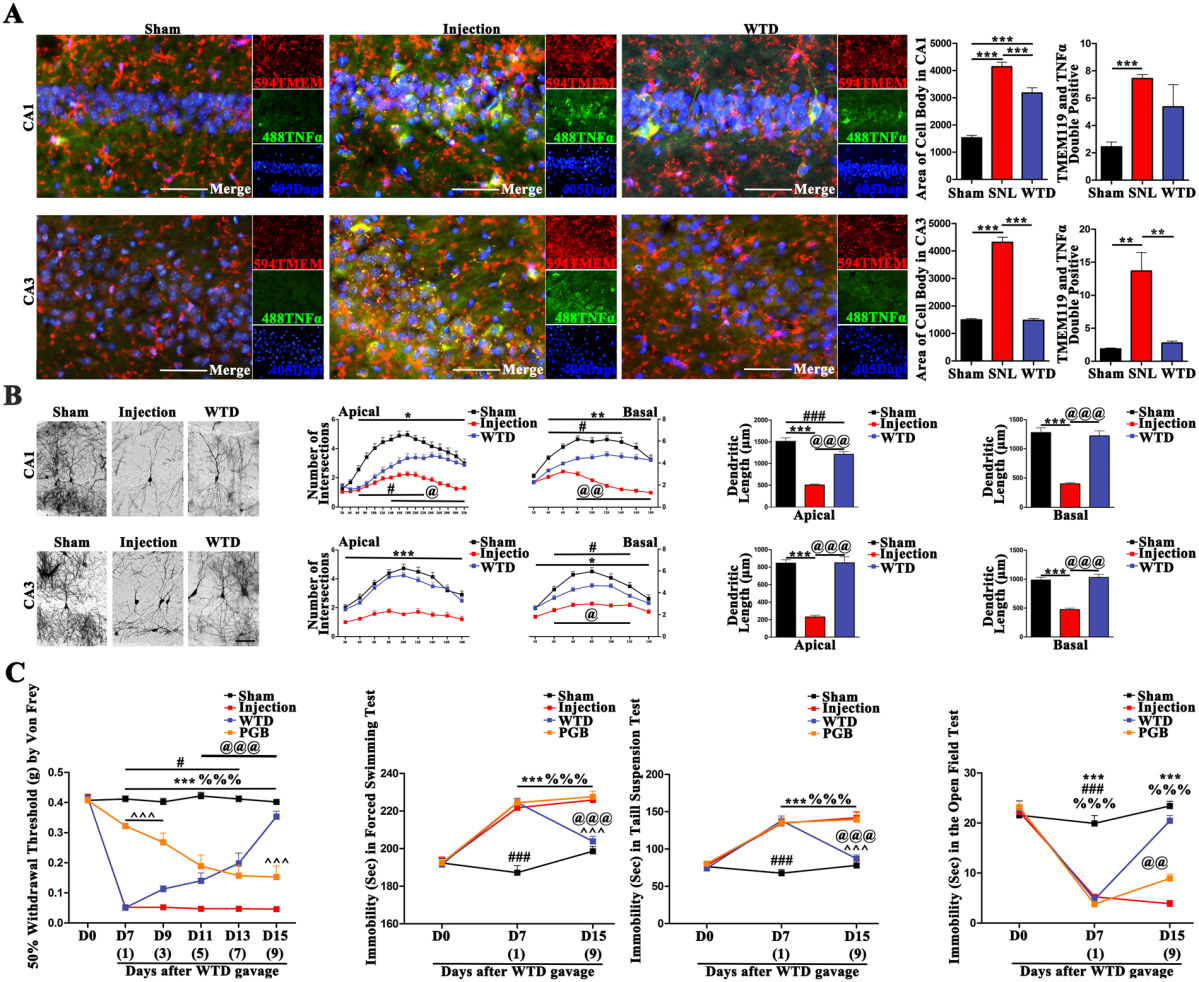
The hippocampal injection of TNFα compromised to the over-activation of microglia, atrophy of hippocampus and the comorbidity of NP syndromes, which were in sum relieved by WTD. (**A**) The activation of microglia (right brain) was quantified in CA1 and CA3 by both the area of cell body and the number of cells double stained by TMEM119 and TNFα (*P < 0.05, **P < 0.01, ***P < 0.001, Mean ± SEM, n = 3 mice/group), scale bar 50 μm. (**B**) The morphological alternations of neurons in CA1 and CA3 were analyzed by Golgi staining (n = 4 mice per group, right side). The figures on the left show the imaging of the entire neurons in Sham/Injection/WTD groups, scale bar 100 μm; the scatter diagrams on the right show the statistical data of the intersections and total length of dendrites on both the apical and basal sides. (*P < 0.05, ***P < 0.001 present the significant differences between the Sham and Injection groups; ^#^P < 0.05, ^###^P < 0.001 present the significant differences between the Sham and WTD groups; ^@@^P < 0.005, ^@@@^P < 0.001 present the significant differences between the Injection and WTD groups, Data are shown as Mean ± SEM, n = 4 mice/group). (**C**) The behavior deficits in Injected mice and the revival by WTD. Grouped data (Mean ± SEM, n = 8 mice/group) show the alternations in pain, depression and anxiety behaviors. In which, ^***^ denotes the significant (P < 0.001) difference between Sham and Injection. ^%%%^ denotes the significant (P < 0.001) difference between Sham and PGB. ^^^^^ denotes the significant (P < 0.001) difference between PGB and WTD. ^@^P < 0.05, ^@@^P < 0.005, ^@@@^P < 0.001 denotes both the significant difference between WTD and Injection. ^###^P < 0.001 denotes both the significant difference between WTD and Sham.

Firstly, the injection induced activation of microglia (Fig. 3A) in CA1 (a) and CA3 (b), which were consolidated by the amplified body size as well as the increased number of the cells double stained with TMEM119 and TNFα as compared to the Sham. In addition, compared to the Injection group, the administration of WTD rescued the amplification of cell bodies in CA3/CA1and the over-expression of TNFα in microglia marked by TMEM119 in CA3 (detailed data were shown in supplementary sheet.3.1).

Secondly, the microglia mediated morphological remodeling of neurons (Fig. 3B), shown by the decreases of the dendritic intersection and length by Golgi staining, were found in CA1 and CA3 as compared to Sham. The protection by WTD, represented by the increases of the dendritic intersection and length as compared with Injection group, were further consolidated (detailed data were shown in supplementary sheet.3.2).

Thirdly, the behavior consequences such as the nociceptive, depressive and anxiety symptoms (Fig. 3C) were assessed by the Von Frey filaments (a), forced swimming/Tail Suspension tests (b) and the open field tests (c). From D7 to D15, significant decreases of the paw withdrawal thresholds, increases of the immobility time and the decreases of the central duration time were found in the injection group as compared to sham, which in sum indicate the development of the stable nociceptive, depressive and anxiety symptoms by injection (detailed data are shown in supplementary sheet.3.3).

The analgesic effects by WTD (Fig. 3C) (a), represented the gradual increases of the paw withdrawal thresholds as compared to the injected ones were found from D11 to D15. Further, the recovery of the nociceptive behaviors, represented by the disappearance of the difference between the WTD and sham groups were validated on D15. For the first-line drug Pregabalin (Fig. 3C) (a), the remission of pain, represented by increases of paw withdrawal thresholds as compared to injected ones, were detected from D7 to D15. However, the existence of the significant differences between the PGB and Sham groups indicate the limited analgesic effects of PGB (detailed data were shown in supplementary sheet.3).

The anti-depression and anxiety effects by WTD (Fig. 3C) (b-c), shown by the decreases of the immobility time in both the forced swimming and tail suspension tests, as well as the increases of the central duration time in the open field tests, were validated on D15 as compared to injected ones. For Pregabalin (Fig. 3C) (b-c), no significant decreases in immobility time nor increases in central duration were founded as compared to SNL, indicating the ineffective of PGB when applied for pain induced mental disorders (detailed data were shown in supplementary sheet.3).

### The consecutive activation of hippocampal microglia offset the neuronal protection and the co-curation by WTD

Given the important roles the hippocampal microglia assumed in the development of NP, we further clarify whether the co-curation by WTD is dependent on the modulation of hippocampal microglia.

**Figure 4:**
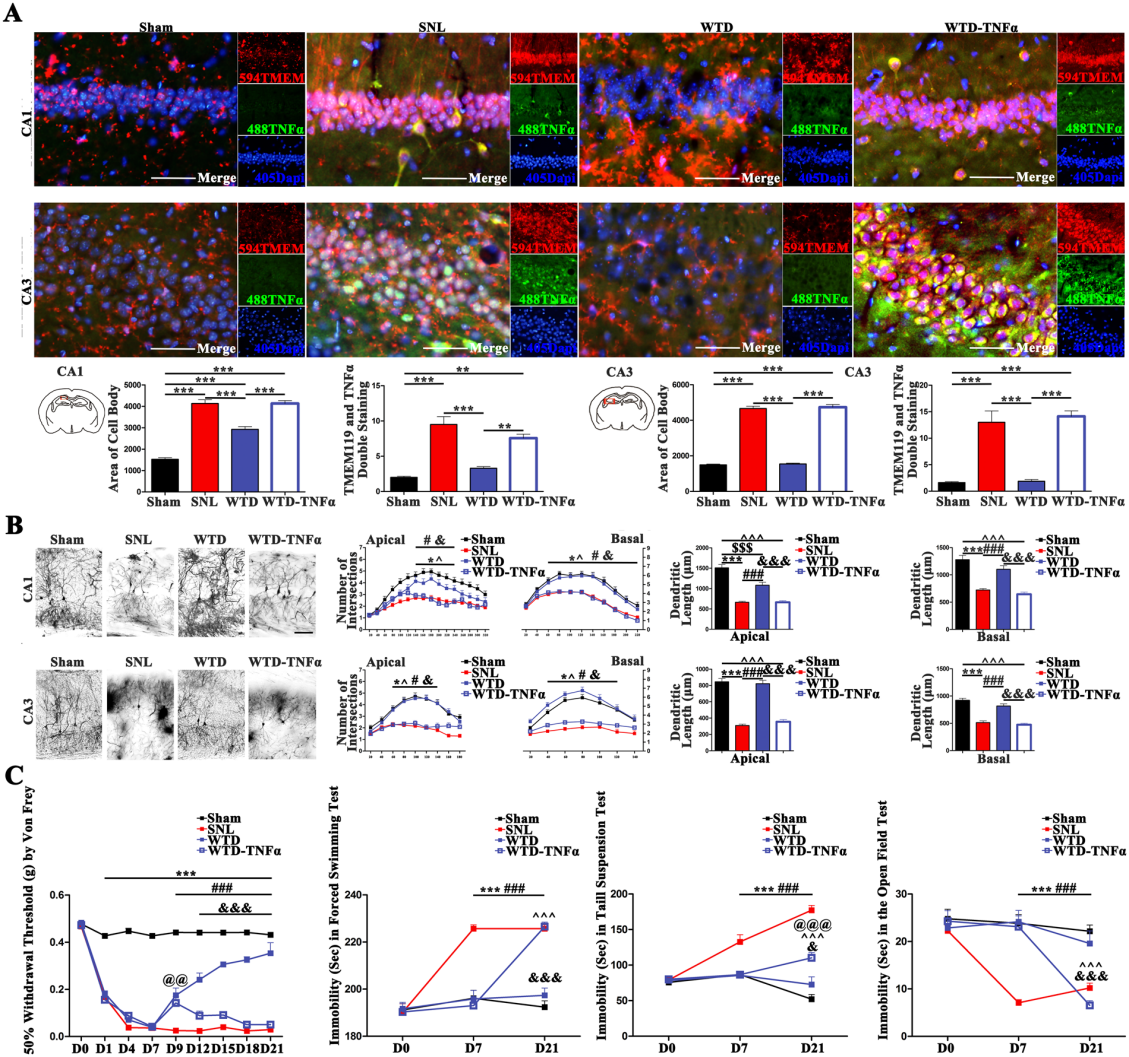
The injection of TNFα in hippocampus abolished the WTD-mediated modulation of hippocampal microglia, rescue of hippocampal neurons and the co-curation for NP behaviors. (**A**) The activation of microglia on the right side was quantified in CA1 and CA3 by both the area of cell body and the number of cells double stained by TMEM119 and TNFα (**P < 0.01, ***P < 0.001, Mean ± SEM, n = 3 mice/group), scale bar 50 μm. (**B**) The morphological alternations of neurons in CA1 and CA3 were analyzed by Golgi staining (n = 4 mice per group, right side). The figures on the left show the imaging of the entire neurons in Sham/SNL/WTD/WTD- TNFα groups, scale bar 100 μm; the scatter diagrams on the right show the statistical data of the intersections and total length of dendrites on both the apical and basal sides. (*P < 0.05, ***P < 0.001 present the significant differences between the Sham and SNL groups; ^$$$^P < 0.001 present the significant differences between the Sham and WTD groups; ^^^P < 0.05, ^^^P < 0.001 present the significant differences between the Sham and WTD- TNFα groups; ^#^P < 0.05, ^###^P < 0.001 present the significant differences between the SNL and WTD groups; ^&^P < 0.05, ^&&&^P < 0.001 present the significant differences between the WTD and WTD- TNFα groups; (Data are shown as Mean ± SEM, n = 4 mice/group). (**C**) The co-curation by WTD was abolished by PGB. Grouped data (Mean ± SEM, n = 8–10 mice/group) show the alternations in pain, depression and anxiety behaviors. In which, ^***^ denotes the significant (P < 0.001) difference between Sham and SNL. ^###^ denotes the significant (P < 0.001) difference between SNL and WTD. ^&&&^denotes the significant (P < 0.001) difference between WTD and WTD- TNFα. ^^^^^ denotes the significant (P < 0.001) difference between WTD and WTD- TNFα. ^@@^ P < 0.005, ^@@@^ P < 0.001 denotes both the significant difference between WTD- TNFα and Sham.

For this aim, purified TNFα protein was injected into the hippocampus of SNL mice, which have undergone the administration of WTD for 9 days (WTD-TNFα group). Despite the significant shrinks of cell body and decreases of cells double stained with TMEM119/TNFα in WTD group as compared to SNL, no significant difference was found between the SNL and WTD- TNFα groups, indicating the consecutive up-regulation of TNFα in hippocampus disabled the inhibition of hippocampal microglia by WTD (Fig. 4A) (detailed data were shown in supplementary sheet.4.1).

The consequences to hippocampal neurons, brought by the consecutive activation of hippocampal microglia in WTD-TNFα group were measured by the Golgi staining. In both CA1 and CA3 region, the failure of protection, represented by the decreases in both the total length and intersections of the dendrites in WTD-TNFα ones, were found as compared to the WTD and Sham groups. The consecutive activation of hippocampal microglia invalidated the neuronal -protective effects of WTD in SNL mice (Fig. 4B) (detailed data were shown in supplementary sheet.4.2).

The consequences to the analgesic, anti-depression and anti-anxiety effects, brought by the sustained activation of hippocampal microglia in WTD-TNFα group were assessed by the Von Frey filaments, forced swimming/tail suspension tests and the open field tests. For WTD-TNFα group, decreases of the paw withdrawal thresholds, increases of the immobility time and the decreases of the central duration time were found as compared to the SNL and WTD groups, which in sum indicate the ineffectiveness of WTD (Fig. 4C) (detailed data were shown in supplementary sheet.4.3).

In conclusion, the modulation of microglia in hippocampus is pivotal for WTD mediated protection of hippocampal neurons and further the nociception, depression and anxiety co-curative effects in SNL.

### The activation of hippocampal microglia led to the imbalance between hippocampal glutamatergic and GABAergic neurons, which were rescued by WTD

**Figure 5:**
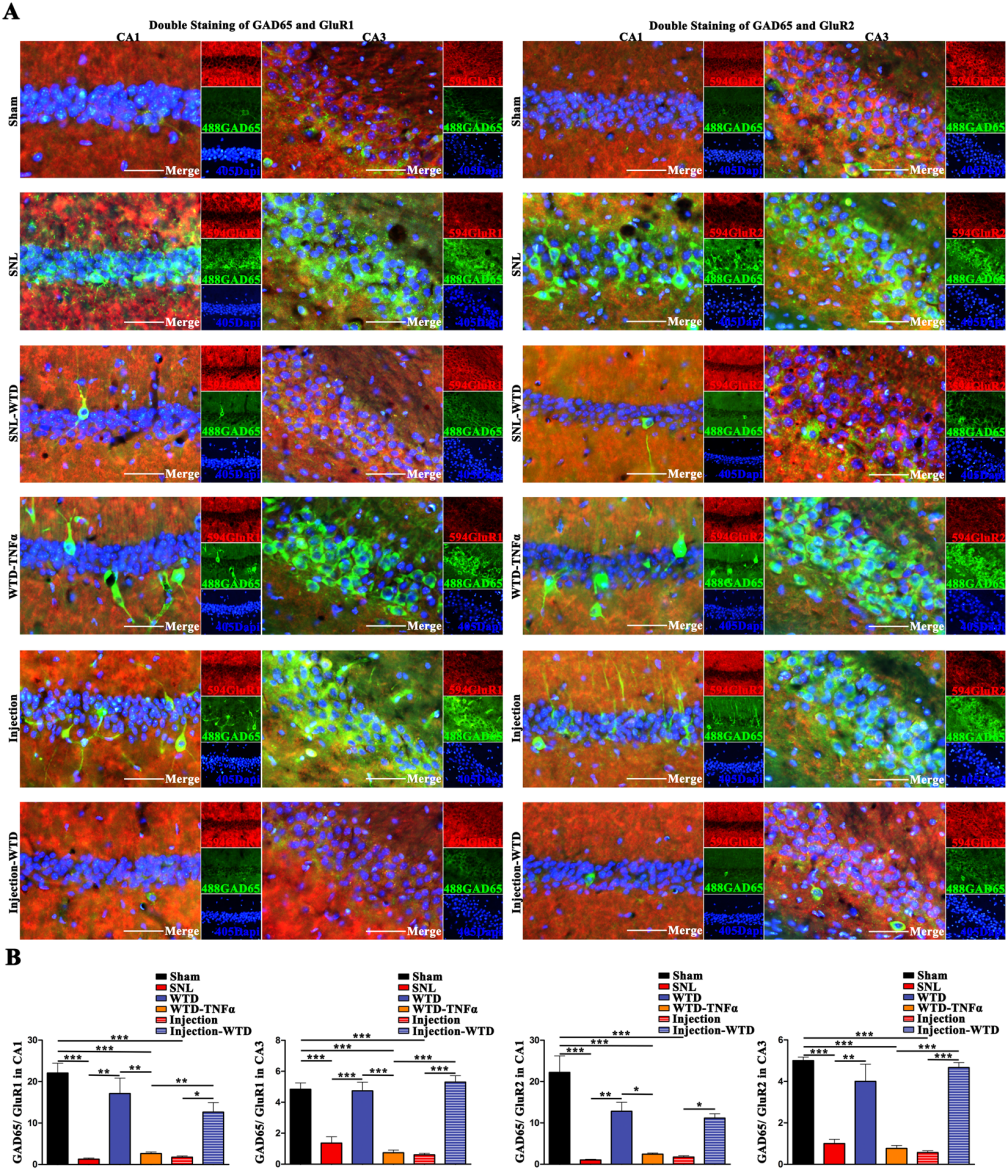
The hippocampal mal-activation of GABAergic and inhibition of glutamatergic neurons in SNL and TNFα injected ones, which were modulated by WTD, and abolished by TNFα. The quantification of the ration between the GAD65 positive and GluR1 positive/GluR2 positive neurons in CA1 and CA3 (right brain) were displayed (**A**) and analyzed (**B**). (Mean ± SEM, n = 3 mice/group), scale bar 50 μm.

To further clarify the harms microglia made to hippocampal neurons and the rescues by WTD, the balance between the glutamatergic and GABAergic neurons in hippocampus were analyzed in the Sham, SNL, WTD, TNFα-Injection, TNFα-Injection-WTD, WTD- TNFα groups by the double staining of glutamatergic neurons by AMPAR1/AMPAR2 and GABAergic neurons by GAD65 (Fig. 5) (detailed data were shown in supplementary sheet.5).

As previously illustrated, significant decreases of the AMPAR1/2 positive and increases of GAD65 positive neurons were found in SNL mice as compared to Sham. In addition, the decreases of AMPAR1/2 positive and increases of GAD65 positive neurons were also validated in the TNFα-Injection ones as compared to the Sham, which indicate that the mal-activation of hippocampal microglia alone is inducible for the imbalance in hippocampus.

Further, the administration of WTD, in both SNL and TNFα-Injection models were found effective in the up-regulation of AMPAR1/2 as well as the down-regulation of GAD65 as compared to the models, indicate hippocampus as the important drug target for WTD in the co-curation of NP. In addition, the consistent decreases of AMPAR1/2 positive and increases of GAD65 positive neurons were found in WTD- TNFα group, indicate the consecutive up-regulation of hippocampal TNFα is resistant for WTD mediated regulation in hippocampus. In sum, the hippocampal microglia are found responsible for the balance between hippocampal glutamatergic and GABAergic neurons. The pathological over-activation of hippocampal microglia is harmful for the balance and further inducible for the comorbidity. WTD, by focusing the modulation of hippocampal microglia, achieved effective pain, depression and anxiety co-curative effects, indicating hippocampal microglia as the important drug target for NP.

## Discussion

### WTD alleviates the long-lasting maladaptive response of hippocampus to the neuropathic pain

Increasing studies show that the maladaptive responses in central nerves system, characterized by the remodeling of the synaptic plasticity, connectivity and circuits, play important roles in the comorbidity of the nociceptive and emotional symptoms in neuropathic pain^28–30^. However, although studies figured out the maladaptive alternations in multiple brain nuclei, the exact roles each brain nuclei assumed in the comorbidity remain unexplained. In previous study, we illustrated the long-lasting degeneration of hippocampal CA3 pyramidal neurons and the WTD mediated rescue in the late stage of SNL operation^27^, represented by the bio-chemical alternations of proteins constructing the synapses, the morphological degeneration of dendrite and dendrite spines assuming the plasticity connections among neurons, as well as the electrophysiological variations of the excitatory potentials.

In this study, by the tracing of the morphological alternations in the limbic system, we described the degeneration of neurons in ACC, BLA, CA1 and CA3 in both the early and late stages after SNL operation. In the early stage, the morphological alternations in ACC, BLA, CA1 and CA3 were found as early as D7, which is consistent with the development of the co-occurred emotional syndromes after the SNL operation. The participation of ACC, BLA and hippocampus in the development of pain induced emotional deficits is consistent with the previous studies^5,7,8,31–33^. Moreover, in the late stage of SNL operation, the long-lasting injures to the limbic system, shown by the permanent morphological degeneration of dendrites, was proven largely limited in hippocampus. The self-recovery in ACC and BLA in the late stage of SNL further indicate that the maintenance of comorbidity in NP is more dependent on hippocampus than ACC or BLA, hinting the rescue of hippocampus as the pivotal drug target in the treatment.

The administration of WTD was found curative for the degeneration of neurons located in the limbic system in SNL mice. In ACC and BLA, WTD accelerated the self-recovery of neurons. In hippocampus, the WTD mediated curation is proven more selective in CA3, which is consistent the more preferred down-regulation of TNFα and up-regulation of brain-derived neurotrophic factor (BDNF) by WTD in CA3^27^. In addition, the unhealed decreases in the apical intersection, apical length and basal length of CA1, is consistent with the un-healed up-regulation of TNFα and down-regulation of BDNF by WTD in CA1. However, the overall recovery of the nociceptive, depressive and anxiety symptoms were proven at this stage in WTD groups, further indicate the un-healed degeneration in CA1 did not influence with co-curation by WTD, clarified the different roles CA1 and CA3 assumed in the maintenance of comorbidity and treatment.

### WTD inhibits the hippocampal neuroinflammation, which is inducible of the comorbidity in NP

Neuroinflammation, especially the mal-activation of microglia in hippocampus, has been widely related with the comorbidity between the nociceptive and emotional syndromes^34^. In the hippocampus of mice with the co-occurrence of the pain-emotion syndromes, we consolidated the mal-activation of microglia and the up-regulation of TNFα. However, no significant over-expression of IL-1β nor the activation of astrocyte were found in SNL mice as compared to sham (data not shown), indicating the selective activation of microglia and the secretion of TNFα may be underlying the pathological mechanism of comorbidity. However, the causal link between the activation of microglia and the damages to neurons remain unclear in NP.

In present study, we consolidated the bio-chemical, morphological and behavioral consequences of the mal-activation of hippocampal microglia. In cultured hippocampal neurons, the long-term incubation with the medium of the over-activated microglia is sufficient to induce the damages to plasticity, which may further lead to the hypofunction of hippocampal neurons under the chronic stresses such as depression or chronic pain^5^. In the SNL and the TNFα hippocampal injected mice, our results gave solid evidence that the hippocampal activation of microglia alone is responsible for the damages to the plasticity of hippocampal pyramidal neurons, and further the induction of the nociceptive, depressive and anxiety behaviors.

Moreover, the inhibition of hippocampal microglia was proven critical for the neuronal protective, as well as the co-curative effects of WTD. The positive evidence was given in co-cultured microglia-hippocampal neurons, as well as the SNL and hippocampal TNFα injection models, in which the administration of WTD significantly inhibited the mal-activation of microglia, rescued the plasticity of hippocampal neurons, and relieved the pain-emotional syndromes. The negative evidence was given by the abolishment of the microglia-protection, neuronal protection and the behavioral modulative effects by the hippocampal injection of TNFα in SNL mice administrated with WTD. On aspects of treatment, we consolidated the pivotal roles hippocampal TNFα assumed in the co-curation as it has been widely reported^13,34^. On aspects of the anti-inflammatory mechanisms of WTD, we illustrated the effective controls in not only the peripheral nerves system and the spinal cord^25,26^, but also the brain nuclei. The consolidation of the immune-regulation in hippocampus, adds to the multi-target mechanism^35–38^ underlying the analgesic effects of WTD in both the acute and chronic pain.

### WTD relieves the imbalance between hippocampal glutamatergic and GABAergic neurons, which is disturbed by the immune activation in hippocampus

In both depression and chronic pain^39^, the hippocampal dysfunction of glutamatergic neurons, represented by the down-regulation of pre-synaptic/post-synaptic glutamate receptors, the degeneration of the length/intersection and the spine density of dendrite, the decreases in mEPSC (miniature excitatory post synaptic current) and LTP (long term potentiation), as well as the abnormal projections to prefrontal cortex and the amygdala have been widely discussed. In present study, we consolidated not only the hypo-function of glutamatergic neurons, but also the TNFα mediated hyper-activation of the hippocampal GABAergic neurons in the hippocampus of both the SNL and TNFα injected mice, which were rescued by WTD.

However, the exact mechanism underlying the differential regulation of glutamatergic and GABAergic neurons by microglia remain unexplored. In the following studies, to clarify the causal links between the differential regulation in hippocampus and the comorbidity in NP, more exact labeling of the interactions among the hippocampal GABAergic/Glutamatergic neurons and microglia, the tracing of the projections in the hyperactivated hippocampal GABAergic neurons will be analyzed in both the SNL and the hippocampal TNFα injected ones with/without the administration of WTD. In addition, to illustrate the molecular mechanism underlying the microglia mediated regulation of glutamatergic and GABAergic neurons, more elaborate quantitative analysis, morphological and electrophysiological analysis will be conducted in the co-culture system of the hippocampal and microglia.

## Conclusion

In this study, we traced the morphological alternations of neurons in the limbic system of SNL mice, figured out the long-lasting atrophy of hippocampus, which was proven induced by the mal-activation of hippocampal microglia and responsible of the maintenance of the comorbidity of pain-emotion syndromes. Moreover, the immune-modulative effects of WTD was consolidated in the hippocampus, proven responsible for the rescue of hippocampal neurons, and found pivotal for the co-curation of the nociceptive and emotional syndromes by WTD.

## Materials and Methods

### Animals

All of the animals used in this study were male adult (8-week old, 26–28 g weight). Mice were kept in the specific pathogen free circumvents, with available food and water, with a 12 hr-light-dark cycle. Before the experiments, the baseline of the nociceptive, depressive and anxiety behaviors were tested to exclude the abnormal ones. The others were divided into experimental groups randomly. In this study, adequate measures were taken to minimize pain or discomfort. The animal experiments were supervised and approved by the Research Ethics Committee of China Academy of Chinese Medical Science, Beijing, China (permission number: 2016–004) and conducted by the trained researchers following the standards in accordance with NIH guidelines.

### The Spinal Nerve Ligation

The spinal nerve ligation (SNL) mice have been widely used in the researches of neuropathic pain^40^. In this study, the SNL mice were constructed as reported^41^. Briefly, after the anesthetization by Isoflurane Inhalation Anesthesia, the L5 spine nerve on the left side was expose and tightly ligated it with a surgical suture. The skin was then washed with normal saline and sutured. In Sham ones, the L5 spine was naked without the following ligation.

### The Injection of purified TNFα in hippocampus

The consecutive 7 days hippocampal injection of TNFα were conducted as follows. The dosage of TNFα (2 ng/day/mice, recombinant mouse TNF-α, ab157351)were fixed^17,20,42^, the exact position of the micro drug delivery trocars (AP: −1.6 mm, R:−2 mm, DV:−2.1 mm relative to bregma and dural surfaces) were located^27^ according to previous studies.

### Drugs

#### Pregabalin (PGB)

According to the guide for the clinical prescription of Pregabalin (Pfizer, J20100102), PGB was dissolved in distilled water to 75 mg/ml, administered 0.3 ml/30 g body weight).

#### Wu-Tou decoction (WTD)

The formula of WTD used in this study is consisted of five herbs (Radix Aconiti/Wu Tou, Herba Ephedrae/Ma Huang, Radix Astragali/Huang Qi, Raidix Paeoniae Alba/Bai Shao and Radix Glycytthizae/Gan Cao, 6:9:9:9:9), which has been normalized by the China Pharmacopoeia standard of quality control^23,43–45^. For the repeatability of the experiments, the herbs were purchased from Beijing Huamiao Chinese Medicine Engineering Development Center (Beijing, China) and authenticated by Professor Dong Zhang, China Academy of Chinese Medical Sciences.

The water extract of WTD was applied in this study, and prepared as follows: Firstly, all the components of WTD were immersed in distilled water for 1 hr (The volume of distilled water is 10 times of the dry weight of herbs in WTD. Secondly, all the components were heated to refluxing for 1.5 h. The supernatant was filtered and stored; Thirdly, distilled water (8 times volume of total dry weight) were added and heated for another 1.5 h refluxing. The supernatant was filtered and stored with the prior supernatant together; Lastly, the supernatant was concentrated to 1.68 g (dry weight of herbs)/mL, sub packaged in tubes, stored in −20 °C. Before the usage, drug was heated to room temperature.

Eleven main components with specific anti-inflammation effects (Benzoylmesaconine, Aconitine, Benzoylhypacoitine, Benzoylaconitine, Hypaconitine, Ephedrine, Calycosin-7-glucoside, Glycyrrhizic acid, Liquiritin, Formononetin, Liquiritigenin) were further used as standards for quality monitoring in our studies^35^.

To exploring the mechanism underlying the pain-emotion co-curation by WTD, the high dose (12.6 g/kg body weight of mice), equivalent to 2 times of the dose clinically prescribed for *rheumatoid arthritis* patients and proven competent for the pain-emotion co-curation^27^, was used in this study. WTD were prescribed to mice as follows: for mice underwent SNL operations with/without the following hippocampal injections of TNFα mice, WTD was administrated from D1 to D21, given by gavage at 9:00 am; for mice after the consecutive 7-day hippocampal injections of TNFα, WTD was administrated from D1 to D9, given by gavage at 9:00 am. In Sham, SNL and Injection groups, equal volume of distilled water was administrated in the same way.

### Behavior tests

#### Mechanical Allodynia

From D0 to D21 after the SNL operations; D0/7 after the initial hippocampal Injections and D1-D9 after the last injections, the Von Frey filaments tests were performed 1 hour after the daily gavage. The detections and analysis of the mechanical allodynia was performed as reported^46^. Briefly, after a 30 min acclimation to the separated cubicles, Von Frey filaments (0.008–4.0 g) tests were conducted 6 times, with 5 min intervals. In each test, the Von Frey filaments were perpendicularly presented to the left paw, held for 5–8 s, with a slight bend of the filament. A positive response is defined as the appearance of an abrupt withdrawal of the paw or by a flinching behavior.

#### Forced swimming test

On D0/7/21 after SNL operations; before and D1/9 after the hippocampal injections, forced swimming test were performed 4-hour after the daily gavage as reported^47,48^. The tests were conducted in a glass cylinder 26 cm in height and 16 cm in diameter containing 14 cm of water at 25 ± 1 °C. After the 1-hour acclimation, the 6 min tests were performed. The first 2 min swimming was just adaption, the last 4 min swimming was the real test, in which the duration of immobility or passive swimming were measured.

#### Tail suspension test

On D0/7/21 after SNL operations; before and D1/9 after the hippocampal injections, forced swimming test were performed 4-hour after the daily gavage as reported^49^. The tests were conducted in a suspension box (55 cm height, 60 cm width, 11.5 cm depth). In the separated compartments (15 cm width/mouse), four mice were tested at the same time. After the 1-hour acclimation, the 6 min tests were performed, in which the first 2 min tailing was just adaption, the last 4 min tailing was the real test. In the 4 min recording, the immobility time was analyzed.

#### Open field test

On D0/7/21 after SNL operations; before and D1/9 after the hippocampal injections, the open field tests were performed 4hrs after the daily gavage. The tests were conducted in a square white test cage, with 50 * 50 cm width, 30 cm height and a border region 8 cm width. After the 1-hour acclimation, mice were placed in the border region of the testing box, facing the wall. During the 4 min test time, the total time mice stayed in the central region was measured.

### Primary hippocampal neuron and microglia

#### Isolation and culture of hippocampal neurons

On D18, two pregnant ICR mice were sacrificed, the hippocampus of brains were dissected in cold phosphate-buffered saline (PBS). The hippocampus was dissected and digested for hippocampal neurons as reported^50^. Neurons were plated on the glass covers (Fisher FIS 12-545-82), 1 × 10^4^ cells/plate.

#### Isolation and culture of microglia

The hippocampus of newborn mice was digested. The separated cells were plated in 6-well plate. After the 14-day incubation, the microglia located on the top layer were digested, centrifuged, resuspended and re-plated on the glass covers (Fisher FIS 12-545-82), 1 × 10^4^ cells/plate^51^.

#### Transfections

To describe the morphological plasticity of hippocampal neurons, 0.8 μg PEGFP-N1 plasmids(Genebank U55762) were transfected into hippocampal neurons by Lipofectamine 2000 (Invitrogen USA) on DIV 12.

#### Dendrite and Spine Assays of hippocampal neurons

To illustrate the influences activated microglia brought to the plasticity of primary hippocampal neurons, the dendrite and spines of hippocampal neurons were analyzed as follows: Firstly, cultured microglia were activated with lipopolysaccharide (LPS) (1 μg/ml) 48 hours before the experiment, treated with WTD (final concentration is 1.25–2.5–5 μg/ml, presenting the low-medium-high doses of WTD) or equivalent volume of PBS 24 hours before the experiment. Secondly, the dendrites and spines of hippocampal neurons were labeled by transfection. Thirdly, the mediums of resting/activated microglia were added to hippocampal neurons, incubated for 12 hours. Lastly, Glycine (final concentration is 200 μM) or equivalent volume of PBS was added and incubated for another 40 minutes. Then, the neurons were fixed, stained and analyzed.

#### Morphological analysis of cultured hippocampal neurons

The dendrites and spines of cultured hippocampal neurons transfected with PEGFP-N1 were stained with primary antibody to EGFP(Earthox E022410 1:200) and secondary antibody (delight Goat anti mouse 488 earthox E032210 1:400) as reported^52^. Z- series Confocal images were obtained with the Olympus FV1000 confocal microscope. Pictures were taken under 100x oil-immersion objective. The stubby, thin and mushroom spines were distinguished as published studies^53^.

#### Other quantification tests

To ensure the credibility of the experiments, all quantification tests were performed from behaviorally tested ones. Brain tissues on the right side were analyzed.

#### Immuno-staining

The 18 μm brain slices were dried at 42 °C, hydrated by PBS, permeabilized and blocked with 5% BSA in PBST(PBS with 0.5% triton) incubated with primary antibody (goat anti-TNFα 1:200 R&D systems AF-410-NA; goat anti-TMEM119 1:200 abcam ab209064; rabbit anti-AMPAR1 1:100 abcam ab183797; rabbit anti AMPAR2 1:600 abcam ab206293, mouse anti-GAD65 1:200 abcam ab26113) at 4 °C overnight. For secondary antibody, delight donkey anti goat 488(earthox E032231 1:200), delight donkey anti rabbit 594(earthox E032421 1:200), delight goat anti mouse 488(earthox E032210 1:400), delight goat anti rabbit 594(earthox E032420 1:400)were used.

#### Golgi-staining

Brains were pre-treated according to the protocol (FD Rapid Golgistain kit). Serial coronal sections (200 μm) were prepared (Leica VT1200 vibratome, Germany) and stained. The pyramidal neurons in ACC, BLA, CA1 and CA3 were photographed, the dendritic length and intersections were analyzed by NeuroJ (plugin of ImageJ NIH) and Sholl.

### Statistical Analyses

One-way ANOVA analyses (followed by Tukey post tests) were used to analyze the difference among groups. The statistical significance was set at P < 0.05. Data in the text were presented as the mean ± SEM. The sample sizes were noted in the appendix of figures.

### Data availability statement

All the primary data supporting this article is available and uploaded with the manuscript.

### Ethical approval and informed consent

All animal experiments were supervised and approved by the Research Ethics Committee of China Academy of Chinese Medical Science, Beijing, China (permission number: 2016-004) and conducted by the trained researchers following the standards in accordance with NIH guidelines. To ensure the credibility of the experiments, all quantification tests were performed from behaviorally tested ones.

## Electronic supplementary material

Supplementary Information
