# Supplementary material for: The Chinese Medicine Wu-Tou Decoction Relieves Neuropathic Pain by Inhibiting Hippocampal Microglia Activation
Source: Sci Rep. 2018 Aug 16;8:12292. doi: 10.1038/s41598-018-30006-7 (PMC6095857; doi:10.1038/s41598-018-30006-7)
Supplement: Supplementary file 1 — Supplementary Information [file 41598_2018_30006_MOESM1_ESM.pdf]

Title: The Chinese Medicine Wu-Tou Decoction Relieves Neuropathic Pain by Inhibiting Hippocampal Microglia Activation.

Authors: Chunyan Zhu1#, Qionghong Xu1#, Zhiyun Mao1, Na Lin1*
Affiliation: 1. Institute of Chinese Materia Medica, China Academy of Chinese Medical Sciences, Beijing 100700, China;
First author: Chunyan Zhu, Qionghong Xu
* Corresponding author: Prof. Na Lin
Address correspondence to: Institute of Chinese Materia Medica, China Academy of Chinese Medical Sciences, No. 16, Nanxiaojie, Dongzhimennei, Beijing 100700, China.
E-mail: linna888@163.com
Phone: +861064014411-2869 Fax: +861064013996

Supplementary figure. 1 The SNL induced morphological alternations in hippocampus and the remission by WTD. (A)-(B) present the morphological alternations on the right side of ACC and BLA (n=4 mice/ group). In (a), the figures on the left side show the imaging of neurons detected on D3/ 7/ 10/ 14/ 18/ 21 in Sham/ SNL /WTD groups, scale bar 100μm; the scatter diagrams on the right show the statistical data of the intersections of dendrites on both the apical and basal sides. In (b), figures show the analyzations of the total length of dendrites on both the apical and basal sides. (*P < 0.05, **P < 0.01, ***P < 0.001 present the significant differences between the Sham and SNL groups; #P < 0.05, ##P < 0.01, ###P < 0.001 present the significant differences between the Sham and WTD groups; &P < 0.05, &&P < 0.01, &&&P < 0.001 present the significant differences between the SNL and WTD groups) (Data are shown as Mean ± SEM).

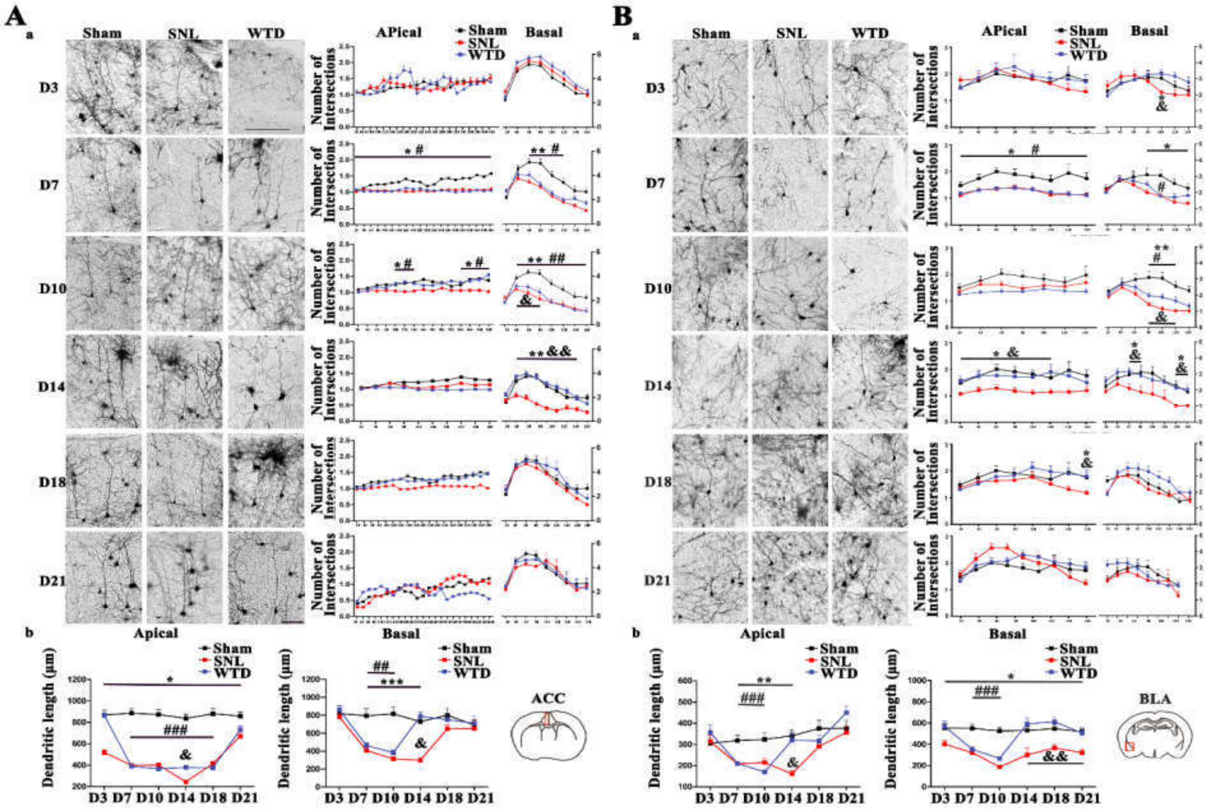

Supplementary data sheet 1.1

The Total length of the dendrites of neurons in ACC

Apical side	Sham			SNL			WTD		
	**Mean**	**SEM**	**N**	**Mean**	**SEM**	**N**	**Mean**	**SEM**	**N**
**D3**	869.1	47.1	30	518.4011	25.91974	61	864.5193	45.0969	59
**D7**	885.7	46.51	30	399.7561	11.16613	78	390.0303	11.6627	76
**D10**	873.8	46.85	30	400.7	11.68	57	365.8	13.78	42
**D14**	836.3	41.19	30	244.4	18.16	23	379.3691	13.30138	74
**D18**	880.2	45.73	30	415.7256	21.56269	31	375.0183	23.28753	16
**D21**	858.2	41.02	30	671.4429	34.74683	21	732.2765	36.69658	30

The Total length of the dendrites of neurons in ACC

Basal side	Sham			SNL			WTD		
	**Mean**	**SEM**	**N**	**Mean**	**SEM**	**N**	**Mean**	**SEM**	**N**
**D3**	819.7	85.08	25	780.8024	38.57319	40	862.6121	44.93413	42
**D7**	794.4	81.21	25	407.9932	19.92106	66	462.6447	28.31425	50
**D10**	815.7	78.83	25	313.7	26.58	30	383.6	31.76	42
**D14**	729.4	78.01	25	299.5	31.38	20	788.3	48.34	39
**D18**	801.4	77.33	25	649.1868	41.10367	20	751.0417	67.06751	16
**D21**	691.6	57.27	25	652.0418	87.47805	11	710.549	79.65509	15

The Total length of the dendrites of neurons in BLA

Apical side	Sham			SNL			WTD		
	**Mean**	**SEM**	**N**	**Mean**	**SEM**	**N**	**Mean**	**SEM**	**N**
**D3**	305.8	24.78	22	314.5215	29.82354	17	356.7745	35.87848	15
**D7**	318.4	24.95	22	208.6183	10.59873	52	210.2447	9.524912	67
**D10**	323.9	29.46	22	215.2	17.9	30	170.6	10.12	65
**D14**	340.7	29.98	22	161.2	14.6	13	319.7	35.43	33
**D18**	375.5	38.4	22	291.202	26.84857	26	316.4409	25.89745	35
**D21**	375.9	38.68	22	356.514	33.19829	28	449.9105	31.54271	30

The Total length of the dendrites of neurons in BLA

Basal side	Sham			SNL			WTD		
	**Mean**	**SEM**	**N**	**Mean**	**SEM**	**N**	**Mean**	**SEM**	**N**
**D3**	553.2	41.56	26	402.0914	34.18863	15	568.6763	49.21918	18
**D7**	551	40.76	26	322.2176	24.7744	39	353.9322	26.83492	61
**D10**	525.7	36.94	26	187.3	13.15	20	266.3	16.39	65
**D14**	530.3	36.84	26	299.9	68.01	12	591.1	58.16	32
**D18**	546.4	42.73	26	366.5951	35.67108	22	611.8619	45.83976	27
**D21**	518.4	35.63	26	322.3702	35.94712	20	505.3703	43.37779	26

The Total length of the dendrites of neurons in CA1

Apical side	Sham			SNL			WTD		
	**Mean**	**SEM**	**N**	**Mean**	**SEM**	**N**	**Mean**	**SEM**	**N**
**D3**	1500	73.77	41	1014.114	34.26218	94	1493.391	49.21793	56
**D7**	1546	72.14	41	659.1836	59.4239	23	590.5604	26.96747	80
**D10**	1559	64.3	41	550	26.94	61	698	41.64	58
**D14**	1481	62.82	41	416.8	21.8	53	661.3	56.29	64
**D18**	1430	57.24	41	583.5616	54.61628	27	826.3201	29.63046	99
**D21**	1519	74.71	41	663.545	23.77564	51	1082.895	70.65735	26

The Total length of the dendrites of neurons in CA1

Basal side	Sham			SNL			WTD		
	**Mean**	**SEM**	**N**	**Mean**	**SEM**	**N**	**Mean**	**SEM**	**N**
**D3**	1100	39.93	44	1105.063	32.87495	80	1233.473	47.84898	53
**D7**	1168	50.38	44	1026.478	65.53818	18	1079.436	40.96967	63
**D10**	1158	51.89	44	1009	38.46	37	1007	37.17	43

**D14**	1211	44.88	44	823.8	40.46	34	1083	43.24	51
**D18**	1222	61.02	44	815.1485	55.93149	23	1014.946	31.96068	82
**D21**	1269	59.5	44	722.8727	27.05834	62	1101.756	67.27679	22

The Total length of the dendrites of neurons in CA3

**Apical side**	**Sham**			**SNL**			**WTD**		
	**Mean**	**SEM**	**N**	**Mean**	**SEM**	**N**	**Mean**	**SEM**	**N**
**D3**	845.3529	40.43434	38	661.1539	44.73502	28	654.9861	52.90111	32
**D7**	845.3529	40.43434	38	390.18	35.08107	32	322.1275	23.07741	54
**D10**	845.3529	40.43434	38	306.6	27.22	33	275.8	27.81	45
**D14**	845.4	40.43	38	280.3	25.31	26	435.3	54.69	22
**D18**	845.3529	40.43434	38	444.1767	40.13475	33	468.7757	70.65599	14
**D21**	845.3529	40.43434	38	309.1978	16.93647	43	822.0101	41.64877	42

The Total length of the dendrites of neurons in CA3

**Basal side**	**Sham**			**SNL**			**WTD**		
	**Mean**	**SEM**	**N**	**Mean**	**SEM**	**N**	**Mean**	**SEM**	**N**
**D3**	950.8	57.8	23	877.6861	45.15229	40	939.2206	55.2579	42
**D7**	971.3	65.98	23	609.2456	52.78819	26	535.3558	35.83024	49
**D10**	977	65.26	23	517.6	38.9	24	569.5	54.03	32
**D14**	965.6	68.9	23	683.5	38.1	22	632.3316	52.73817	19
**D18**	1008	66.91	23	653.8129	49.75276	23	571.3798	50.0676	17
**D21**	919.8	61.01	23	513.6967	30.18695	35	816.9096	39.8946	37

Supplementary data sheet 1.2
The intersections of the dendrites of neurons in ACC

Apical side		Sham			SNL			WTD		
		Mean	SEM	N	Mean	SEM	N	Mean	SEM	N
D3	20	1.071429	0.071429	42	1.066667	0.037605	45	1.070423	0.030581	71
	40	1.119048	0.060987	42	1.088889	0.053392	45	1.028169	0.019776	71
	60	1.214286	0.105239	42	1.244444	0.090763	45	1.028169	0.019776	71
	80	1.071429	0.071429	42	1.222222	0.077126	45	1.066667	0.032475	60
	100	1.119048	0.060987	42	1.444444	0.112616	45	1.25	0.084456	60
	120	1.214286	0.105239	42	1.377778	0.096864	45	1.533333	0.131198	60
	140	1.238095	0.095238	42	1.355556	0.085017	45	1.533333	0.122282	60
	160	1.238095	0.095238	42	1.288889	0.068329	45	1.75	0.149623	60
	180	1.285714	0.10385	42	1.244444	0.064788	45	1.7	0.128661	60
	200	1.309524	0.110369	42	1.177778	0.06582	45	1.058824	0.033276	51
	220	1.404762	0.127786	42	1.2	0.07521	45	1.196078	0.084108	51
	240	1.333333	0.111305	42	1.133333	0.051247	45	1.352941	0.107825	51
	260	1.309524	0.115511	42	1.2	0.07521	45	1.392157	0.115736	51
	280	1.190476	0.077998	42	1.155556	0.06321	45	1.54902	0.119849	51
	300	1.238095	0.074739	42	1.4	0.139986	45	1.490196	0.116728	51
	320	1.404762	0.127786	42	1.348837	0.114718	43	1.0625	0.035308	48
	340	1.428571	0.103048	42	1.348837	0.093438	43	1.166667	0.08063	48
	360	1.380952	0.101969	42	1.425	0.123452	40	1.3125	0.09945	48
	380	1.428571	0.097249	42	1.405405	0.119178	37	1.333333	0.108721	48
	400	1.428571	0.103048	42	1.378378	0.11198	37	1.479167	0.111365	48
	420	1.52381	0.114247	42	1.515152	0.131435	33	1.395833	0.092921	48
D7	20	1.071429	0.071429	42	1.013333	0.013333	75	1.070423	0.030581	71
	40	1.119048	0.060987	42	1.08	0.08	75	1.028169	0.019776	71
	60	1.214286	0.105239	42	1.053333	0.041992	75	1.028169	0.019776	71
	80	1.238095	0.095238	42	1.04	0.029653	75	1.028169	0.019776	71
	100	1.238095	0.095238	42	1.053333	0.026121	75	1.014084	0.014085	71
	120	1.285714	0.10385	42	1.026667	0.018728	75	1.028169	0.019776	71
	140	1.309524	0.110369	42	1.04	0.029653	75	1.070423	0.030581	71
	160	1.404762	0.127786	42	1.013333	0.013333	75	1.126761	0.044539	71
	180	1.333333	0.111305	42	1.026667	0.018728	75	1.098592	0.035631	71
	200	1.309524	0.115511	42	1.040541	0.023083	74	1.112676	0.037793	71
	220	1.190476	0.077998	42	1.027397	0.019238	73	1.058824	0.035571	68
	240	1.238095	0.074739	42	1.028169	0.019776	71	1.048387	0.027475	62
	260	1.404762	0.127786	42	1.073529	0.038154	68	1.052632	0.029839	57
	280	1.428571	0.103048	42	1.079365	0.034329	63	1.037037	0.025941	54
	300	1.380952	0.101969	42	1.071429	0.043073	56	1	0	48
	320	1.428571	0.097249	42	1.090909	0.046945	55	1.022727	0.022727	44
	340	1.428571	0.103048	42	1.068182	0.038438	44	1.026316	0.026316	38
	360	1.52381	0.114247	42	1.058824	0.040959	34	1.060606	0.04218	33
	380	1.47619	0.109046	42	1.074074	0.051361	27	1.034483	0.034483	29
	400	1.571429	0.136927	42	1.1	0.068825	20	1.05	0.05	20
D10	20	1.071429	0.071429	42	1.018182	0.018182	55	1	0	38
	40	1.119048	0.060987	42	1.036364	0.025474	55	1.052632	0.03671	38
	60	1.214286	0.105239	42	1.036364	0.025474	55	1.162162	0.072633	37
	80	1.238095	0.095238	42	1.054545	0.040354	55	1.135135	0.056978	37
	100	1.238095	0.095238	42	1.036364	0.025474	55	1.27027	0.167335	37
	120	1.285714	0.10385	42	1.018182	0.018182	55	1.324324	0.116596	37
	140	1.309524	0.110369	42	1.036364	0.025474	55	1.27027	0.092095	37
	160	1.404762	0.127786	42	1.090909	0.059587	55	1.216216	0.068611	37
	180	1.333333	0.111305	42	1.037037	0.025941	54	1.216216	0.068611	37
	200	1.309524	0.115511	42	1.12963	0.079509	54	1.277778	0.102439	36
	220	1.190476	0.077998	42	1.055556	0.031464	54	1.352941	0.110761	34
	240	1.238095	0.074739	42	1.057692	0.032649	52	1.3125	0.104703	32
	260	1.404762	0.127786	42	1.061224	0.034604	49	1.357143	0.11745	28

	280	1.428571	0.103048	42	1.066667	0.037605	45	1.4	0.129099	25
	300	1.380952	0.101969	42	1.027027	0.027027	37	1.55	0.169752	20
	20	1.071429	0.071429	42	1.043478	0.043478	23	1.026667	0.026667	75
	40	1.119048	0.060987	42	1.086957	0.060074	23	1.08	0.08	75
	60	1.214286	0.105239	42	1.217391	0.087939	23	1.08	0.08	75
	80	1.238095	0.095238	42	1.130435	0.071802	23	1.08	0.08	75
D14	100	1.238095	0.095238	42	1.047619	0.047619	21	1.013333	0.013333	75
	120	1.285714	0.10385	42	1.095238	0.065638	21	1	0	75
	140	1.309524	0.110369	42	1.117647	0.080547	17	1	0	74
	160	1.404762	0.127786	42	1.214286	0.154727	14	1	0	73
	180	1.333333	0.111305	42	1.153846	0.153846	13	1.027778	0.019503	72
	200	1.309524	0.115511	42	1.166667	0.112367	12	1.014286	0.014286	70
	20	1.071429	0.071429	42	1	0	32	1.035714	0.035714	28
	40	1.119048	0.060987	42	1	0	32	1.035714	0.035714	28
	60	1.214286	0.105239	42	1.03125	0.03125	32	1.107143	0.059524	28
	80	1.238095	0.095238	42	1.0625	0.0625	32	1.178571	0.073707	28
	100	1.238095	0.095238	42	1.09375	0.06897	32	1.214286	0.078967	28
	120	1.285714	0.10385	42	1.125	0.07446	32	1.285714	0.113356	28
	140	1.309524	0.110369	42	1	0	32	1.321429	0.192818	28
	160	1.404762	0.127786	42	1	0	32	1.25	0.132388	28
	180	1.333333	0.111305	42	1.03125	0.03125	32	1.25	0.121988	28
D18	200	1.309524	0.115511	42	1.0625	0.0625	32	1.285714	0.124479	28
	220	1.190476	0.077998	42	1.09375	0.06897	32	1.296296	0.104252	27
	240	1.238095	0.074739	42	1.125	0.07446	32	1.296296	0.117121	27
	260	1.404762	0.127786	42	1.09375	0.06897	32	1.307692	0.133235	26
	280	1.428571	0.103048	42	1.09375	0.06897	32	1.346154	0.123317	26
	300	1.380952	0.101969	42	1.09375	0.06897	32	1.291667	0.127393	24
	320	1.428571	0.097249	42	1.09375	0.052351	32	1.363636	0.168331	22
	340	1.428571	0.103048	42	1.064516	0.044853	31	1.473684	0.177184	19
	360	1.52381	0.114247	42	1.133333	0.079269	30	1.388889	0.164474	18
	380	1.47619	0.109046	42	1.034483	0.034483	29	1.5	0.182574	16
	20	1.071429	0.071429	42	1	0	22	1.125	0.07446	32
	40	1.119048	0.060987	42	1	0	22	1.3125	0.145063	32
	60	1.214286	0.105239	42	1.090909	0.062733	22	1.375	0.16033	32
	80	1.238095	0.095238	42	1.227273	0.091449	22	1.375	0.140204	32
	100	1.238095	0.095238	42	1.227273	0.091449	22	1.4375	0.141546	32
	120	1.285714	0.10385	42	1.318182	0.137799	22	1.375	0.16033	32
	140	1.309524	0.110369	42	1.318182	0.137799	22	1.25	0.134704	32
	160	1.404762	0.127786	42	1.363636	0.140276	22	1.375	0.147219	32
	180	1.333333	0.111305	42	1.454545	0.157459	22	1.46875	0.167912	32
	200	1.309524	0.115511	42	1.454545	0.157459	22	1.46875	0.167912	32
	220	1.190476	0.077998	42	1.409091	0.125858	22	1.46875	0.200726	32
D21	240	1.238095	0.074739	42	1.363636	0.123888	22	1.34375	0.123861	32
	260	1.404762	0.127786	42	1.318182	0.121077	22	1.25	0.109985	32
	280	1.428571	0.103048	42	1.380952	0.12866	21	1.258065	0.092373	31
	300	1.380952	0.101969	42	1.47619	0.148309	21	1.387097	0.200416	31
	320	1.428571	0.097249	42	1.578947	0.176314	19	1.16129	0.067151	31
	340	1.428571	0.103048	42	1.611111	0.2444	18	1.225806	0.076337	31
	360	1.52381	0.114247	42	1.666667	0.213896	18	1.266667	0.09509	30
	380	1.47619	0.109046	42	1.647059	0.19061	17	1.285714	0.101015	28
	400	1.571429	0.136927	42	1.529412	0.193984	17	1.307692	0.107692	26
	420	1.487805	0.105472	41	1.5	0.182574	16	1.291667	0.094776	24
	440	1.575	0.112589	40	1.538462	0.183114	13	1.25	0.09934	20
	460	1.6	0.124347	35	1.5	0.230283	12	1.176471	0.095305	17

The intersections of the dendrites of neurons in ACC

Basal side	Sham			SNL			WTD		
	Mean	SEM	N	Mean	SEM	N	Mean	SEM	N

	20	2.189189	0.196966	37	2.883721	0.191772	43	2.452381	0.196374	42
	40	4.540541	0.261649	37	4.744186	0.197136	43	5.238095	0.212309	42
	60	5.108108	0.26504	37	5.395349	0.260206	43	5.642857	0.220393	42
**D3**	80	5	0.371669	37	5.238095	0.272233	42	5.714286	0.221613	42
	100	4	0.354114	36	4.571429	0.321767	42	4.976191	0.27997	42
	120	3.4375	0.375504	32	3.9	0.37348	40	4.375	0.233939	40
	140	2.741935	0.373786	31	3.138889	0.324044	36	3.384615	0.261356	39
	160	2.653846	0.327895	26	2.533333	0.228606	30	2.916667	0.262391	36
	20	2.189189	0.196966	37	2.741935	0.147131	62	2.708333	0.176044	48
	40	4.540541	0.261649	37	3.758065	0.171603	62	4.083333	0.214363	48
	60	5.108108	0.26504	37	3.467742	0.171653	62	4.041667	0.23625	48
**D7**	80	5	0.371669	37	3.068965	0.159138	58	3.255319	0.253501	47
	100	4	0.354114	36	2.309091	0.155424	55	2.538461	0.23764	39
	120	3.4375	0.375504	32	1.820513	0.190432	39	2	0.217597	33
	140	2.741935	0.373786	31	1.517241	0.153936	29	2.095238	0.291995	21
	160	2.653846	0.327895	26	1.117647	0.080547	17	1.714286	0.220603	14
	20	2.189189	0.196966	37	2.655172	0.254399	29	2.162162	0.162162	37
	40	4.540541	0.261649	37	3.448276	0.265348	29	3.783784	0.239291	37
	60	5.108108	0.26504	37	3.115385	0.295228	26	3.675676	0.260094	37
**D10**	80	5	0.371669	37	2.523809	0.328192	21	3.2	0.303481	35
	100	4	0.354114	36	2.2	0.296005	15	2.193548	0.214623	31
	120	3.4375	0.375504	32	2	0.327327	8	1.863636	0.211114	22
	140	2.741935	0.373786	31	1.6	0.4	5	1.4375	0.203485	16
	160	2.653846	0.327895	26	1.333333	0.333333	3	1.363636	0.278722	11
	20	2.189189	0.196966	37	2.380952	0.223353	21	3.025641	0.239453	39
	40	4.540541	0.261649	37	2.95	0.366168	20	5.051282	0.254378	39
	60	5.108108	0.26504	37	2.684211	0.333795	19	5.358974	0.329575	39
	80	5	0.371669	37	1.941176	0.249567	17	4.948718	0.332554	39
**D14**	100	4	0.354114	36	1.466667	0.191899	15	4.263158	0.286451	38
	120	3.4375	0.375504	32	1.2	0.133333	10	3.885714	0.377456	35
	140	2.741935	0.373786	31	1.5	0.288675	4	3.46875	0.37563	32
	160	2.653846	0.327895	26	1.333333	0.333333	3	2.6	0.305505	30
	180	2.681818	0.368608	22	1	0	2	1.961538	0.263005	26
	20	2.189189	0.196966	37	2.571429	0.244671	21	2.6	0.362531	15
	40	4.540541	0.261649	37	4.380952	0.27147	21	4.666667	0.454257	15
	60	5.108108	0.26504	37	4.714286	0.25017	21	4.933333	0.520683	15
	80	5	0.371669	37	4.380952	0.319793	21	4.866667	0.584455	15
**D18**	100	4	0.354114	36	3.904762	0.283723	21	4.6	0.653197	15
	120	3.4375	0.375504	32	3.105263	0.214868	19	4.285714	0.658241	14
	140	2.741935	0.373786	31	2.5	0.258831	18	3.071429	0.507789	14
	160	2.653846	0.327895	26	1.857143	0.231031	14	2.363636	0.387831	11
	180	2.681818	0.368608	22	1.333333	0.166667	9	1.875	0.350382	8
	20	2.189189	0.196966	37	2.181818	0.295979	11	2.466667	0.376281	15
	40	4.540541	0.261649	37	4	0.26968	11	4.466667	0.38873	15
	60	5.108108	0.26504	37	4.272727	0.383546	11	4.6	0.411733	15
	80	5	0.371669	37	4.090909	0.475864	11	4.666667	0.464621	15
**D21**	100	4	0.354114	36	4.545455	0.623241	11	4.266667	0.462567	15
	120	3.4375	0.375504	32	4	0.62361	9	3.6	0.485994	15
	140	2.741935	0.373786	31	2.857143	0.594762	7	3	0.627646	12
	160	2.653846	0.327895	26	2.142857	0.340068	7	2.4	0.686375	10
	180	2.681818	0.368608	22	2.5	0.645497	4	2.285714	0.837067	7

Supplementary data sheet 1.3

The intersections of the dendrites of neurons in BLA

Apical side		Sham			SNL			WTD		
		Mean	SEM	N	Mean	SEM	N	Mean	SEM	N
D3	20	1.483871	0.138121	31	1.764706	0.182495	17	1.466667	0.191899	15
	40	1.741935	0.166996	31	1.823529	0.196201	17	1.866667	0.236375	15
	60	2	0.179605	31	2.176471	0.230653	17	2.133333	0.255728	15
	80	1.903226	0.204018	31	1.941176	0.181306	17	2.266667	0.452155	15
	100	1.806452	0.169879	31	1.823529	0.176471	17	1.928571	0.195113	14
	120	1.68	0.197653	25	1.642857	0.199095	14	1.833333	0.241	12
	140	1.952381	0.341399	21	1.416667	0.228908	12	1.8	0.249444	10
	160	1.75	0.214087	16	1.333333	0.210819	6	1.7	0.260342	10
D7	20	1.483871	0.138121	31	1.117647	0.045565	51	1.190476	0.063428	63
	40	1.741935	0.166996	31	1.313725	0.076646	51	1.301587	0.066493	63
	60	2	0.179605	31	1.352941	0.073156	51	1.380952	0.073074	63
	80	1.903226	0.204018	31	1.416667	0.077833	48	1.387097	0.07749	62
	100	1.806452	0.169879	31	1.333333	0.07785	45	1.327586	0.079274	58
	120	1.68	0.197653	25	1.138889	0.058456	36	1.22	0.082264	50
	140	1.952381	0.341399	21	1.153846	0.07216	26	1.166667	0.093435	36
	160	1.75	0.214087	16	1.136364	0.074887	22	1.117647	0.080547	17
D10	20	1.483871	0.138121	31	1.333333	0.099808	30	1.22973	0.065385	74
	40	1.741935	0.166996	31	1.6	0.140606	30	1.30137	0.069475	73
	60	2	0.179605	31	1.6	0.113462	30	1.347222	0.068973	72
	80	1.903226	0.204018	31	1.461538	0.126865	26	1.333333	0.064002	63
	100	1.806452	0.169879	31	1.565217	0.15175	23	1.431373	0.075433	51
	120	1.68	0.197653	25	1.529412	0.174002	17	1.351351	0.088499	37
	140	1.952381	0.341399	21	1.666667	0.235702	9	1.333333	0.125988	21
D14	20	1.483871	0.138121	31	1.071429	0.071429	14	1.558824	0.169886	34
	40	1.741935	0.166996	31	1.214286	0.113804	14	1.764706	0.198294	34
	60	2	0.179605	31	1.285714	0.125294	14	1.764706	0.198294	34
	80	1.903226	0.204018	31	1.181818	0.121967	11	1.71875	0.175255	32
	100	1.806452	0.169879	31	1.111111	0.111111	9	1.703704	0.183468	27
	120	1.68	0.197653	25	1.142857	0.142857	7	1.894737	0.274605	19
	140	1.952381	0.341399	21	1.142857	0.142857	7	1.75	0.193649	16
	160	1.75	0.214087	16	1.2	0.2	5	1.5	0.203	14
D18	20	1.483871	0.138121	31	1.37037	0.108711	27	1.297297	0.093841	37
	40	1.741935	0.166996	31	1.592593	0.133539	27	1.513514	0.132098	37
	60	2	0.179605	31	1.615385	0.136741	26	1.783784	0.182307	37
	80	1.903226	0.204018	31	1.653846	0.156326	26	1.837838	0.191852	37
	100	1.806452	0.169879	31	1.76	0.20232	25	2.125	0.204535	32
	120	1.68	0.197653	25	1.5	0.147442	24	1.96875	0.212912	32
	140	1.952381	0.341399	21	1.318182	0.121077	22	1.892857	0.207908	28
	160	1.75	0.214087	16	1.176471	0.095305	17	1.8	0.236198	20
D21	20	1.483871	0.138121	31	1.555556	0.154083	27	1.310345	0.100534	29
	40	1.741935	0.166996	31	2.148148	0.204673	27	1.896552	0.134502	29
	60	2	0.179605	31	2.555556	0.17158	27	2.034483	0.152829	29
	80	1.903226	0.204018	31	2.555556	0.187451	27	2.068965	0.139766	29
	100	1.806452	0.169879	31	2.185185	0.20698	27	2.310345	0.17266	29
	120	1.68	0.197653	25	2	0.294245	22	2.214286	0.208021	28
	140	1.952381	0.341399	21	1.882353	0.319494	17	2.037037	0.216693	27
	160	1.75	0.214087	16	1.461538	0.183114	13	1.958333	0.194854	24
	180	1.714286	0.265789	14	1.222222	0.146986	9	1.85	0.181731	20

The intersections of the dendrites of neurons in BLA

Basal side	Sham			SNL			WTD		
	Mean	SEM	N	Mean	SEM	N	Mean	SEM	N
20	2.227273	0.254244	22	2.571429	0.291157	14	1.947368	0.222607	19
40	2.727273	0.309879	22	3.142857	0.31198	14	2.684211	0.230086	19

	60	2.954545	0.36323	22	3.214286	0.260569	14	2.947368	0.222607	19
**D3**	80	3.090909	0.394272	22	2.857143	0.274505	14	3.263158	0.263158	19
	100	3.045455	0.380688	22	2.153846	0.191021	13	3.315789	0.254235	19
	120	2.545455	0.313666	22	2	0.333333	10	3.157895	0.256645	19
	140	2.263158	0.294612	19	2	0.365148	6	2.789474	0.311373	19
	20	2.227273	0.254244	22	2.125	0.16033	32	2	0.104333	53
	40	2.727273	0.309879	22	2.8125	0.197961	32	2.867924	0.132076	53
	60	2.954545	0.36323	22	2.483871	0.190477	31	2.711539	0.165539	52
**D7**	80	3.090909	0.394272	22	2	0.160644	31	2.5	0.180579	46
	100	3.045455	0.380688	22	1.791667	0.18037	24	1.777778	0.178668	36
	120	2.545455	0.313666	22	1.4	0.152177	20	1.714286	0.204817	28
	140	2.263158	0.294612	19	1.333333	0.188025	12	1.8125	0.261705	16
	20	2.227273	0.254244	22	1.894737	0.130109	19	2.037037	0.121034	54
	40	2.727273	0.309879	22	2.473684	0.117688	19	2.685185	0.153742	54
	60	2.954545	0.36323	22	2.0625	0.192976	16	2.471698	0.179912	53
**D10**	80	3.090909	0.394272	22	1.4	0.163299	10	1.96	0.151078	50
	100	3.045455	0.380688	22	1.125	0.125	8	1.861111	0.155087	36
	120	2.545455	0.313666	22	1	0	2	1.625	0.239357	16
	140	2.263158	0.294612	19	1	0	1	1.3	0.213438	10
	20	2.227273	0.254244	22	1.909091	0.211254	11	2.580645	0.226114	31
	40	2.727273	0.309879	22	2.363636	0.309625	11	3.096774	0.228858	31
	60	2.954545	0.36323	22	2.1	0.276887	10	3.16129	0.227642	31
	80	3.090909	0.394272	22	1.875	0.398098	8	2.9	0.236595	30
**D14**	100	3.045455	0.380688	22	1.75	0.75	4	2.62069	0.278162	29
	120	2.545455	0.313666	22	1.5	0.5	2	2.5	0.275839	24
	140	2.263158	0.294612	19	1	0	1	2.130435	0.283292	23
	160	1.882353	0.240746	17	1	0	1	2.052632	0.310383	19
	20	2.727273	0.309879	22	1.956522	0.193555	23	1.875	0.166499	32
	40	2.954545	0.36323	22	2.956522	0.23922	23	3.1875	0.255731	32
	60	3.090909	0.394272	22	3.043478	0.277469	23	3.533333	0.317075	30
	80	3.045455	0.380688	22	2.636364	0.267629	22	3.482759	0.338865	29
**D18**	100	2.545455	0.313666	22	2.190476	0.263846	21	3.307692	0.371149	26
	120	2.263158	0.294612	19	1.947368	0.222607	19	2.96	0.353459	25
	140	1.882353	0.240746	17	1.785714	0.280865	14	2.666667	0.278887	21
	160	1.428571	0.250588	14	2	0.422577	8	2	0.241825	19
	180	1.545455	0.312283	11	1.428571	0.297381	7	2	0.246183	12
	20	2.227273	0.254244	22	2.190476	0.177537	21	3.16	0.268825	25
	40	2.727273	0.309879	22	2.523809	0.190476	21	3.28	0.273983	25
	60	2.954545	0.36323	22	2.761905	0.291995	21	3.28	0.308329	25
	80	3.090909	0.394272	22	2.5	0.28562	20	2.958333	0.33772	24
**D21**	100	3.045455	0.380688	22	2.210526	0.346907	19	2.478261	0.287208	23
	120	2.545455	0.313666	22	2.125	0.385951	16	2.052632	0.235376	19
	140	2.263158	0.294612	19	2.2	0.663325	10	1.875	0.239357	16
	160	1.882353	0.240746	17	1.25	0.25	4	1.916667	0.148647	12

Supplementary data sheet 1.4
The intersections of the dendrites of neurons in CA1

Apical side		Sham			SNL			WTD		
		Mean	SEM	N	Mean	SEM	N	Mean	SEM	N
	20	1.235294	0.103923	34	1.287234	0.068688	94	1.460526	0.111514	76
	40	1.676471	0.182675	34	1.797872	0.115767	94	2.263158	0.170004	76
	60	2.558824	0.283822	34	2.414894	0.134786	94	2.934211	0.205482	76
	80	3.411765	0.295895	34	3	0.164305	94	3.815789	0.23072	76
	100	4.029412	0.320108	34	3.638298	0.185157	94	4.263158	0.232732	76
	120	4.441176	0.330265	34	3.914894	0.170898	94	4.802631	0.221652	76
	140	4.617647	0.304496	34	4.138298	0.175483	94	5.236842	0.20076	76
	160	4.882353	0.320885	34	3.957447	0.170399	94	5.526316	0.199538	76
	180	4.941176	0.24597	34	3.755319	0.15623	94	5.355263	0.207495	76
D3	200	4.647059	0.256406	34	3.623656	0.16029	93	4.921052	0.206698	76
	220	4.441176	0.319288	34	3.26087	0.140701	92	4.776316	0.21651	76
	240	4.151515	0.31116	33	2.755556	0.129641	90	4.27027	0.208392	74
	260	3.96875	0.346307	32	2.511628	0.147709	86	3.816901	0.197886	71
	280	3.741935	0.30398	31	2.291667	0.160005	72	3.5	0.197006	66
	300	3.483871	0.34037	31	2.166667	0.156166	60	2.9	0.195573	60
	320	3	0.290957	28	2.190476	0.187403	42	2.673077	0.224316	52
	340	2.48	0.295071	25	1.882353	0.15664	34	2.204545	0.188405	44
	360	2.315789	0.342443	19	1.84	0.228619	25	2.09375	0.18708	32
	380	2.583333	0.468045	12	1.444444	0.201663	18	1.909091	0.217314	22
	20	1.235294	0.103923	34	1.217391	0.087939	23	1.441558	0.087678	77
	40	1.676471	0.182675	34	1.304348	0.116517	23	1.701299	0.121318	77
	60	2.558824	0.283822	34	1.565217	0.138114	23	1.987013	0.137716	77
	80	3.411765	0.295895	34	1.695652	0.15948	23	2.168831	0.146037	77
	100	4.029412	0.320108	34	1.913043	0.197944	23	2.532468	0.17884	77
	120	4.441176	0.330265	34	2.478261	0.265764	23	2.701299	0.193949	77
	140	4.617647	0.304496	34	2.782609	0.307717	23	2.454545	0.151936	77
D7	160	4.882353	0.320885	34	2.782609	0.344101	23	2.118421	0.154428	76
	180	4.941176	0.24597	34	2.863636	0.379653	22	2.085714	0.166243	70
	200	4.647059	0.256406	34	2.736842	0.313834	19	2.047619	0.162275	63
	220	4.441176	0.319288	34	2.333333	0.333333	18	1.775862	0.107333	58
	240	4.151515	0.31116	33	2.058824	0.234373	17	1.755102	0.125528	49
	260	3.96875	0.346307	32	1.625	0.201556	16	1.72093	0.142457	43
	280	3.741935	0.30398	31	1.785714	0.299791	14	1.8125	0.158353	32
	300	3.483871	0.34037	31	1.2	0.133333	10	1.551724	0.117299	29
	20	1.235294	0.103923	34	1.21875	0.060867	64	1.301887	0.09929	53
	40	1.676471	0.182675	34	1.375	0.098349	64	1.698113	0.173717	53
	60	2.558824	0.283822	34	1.609375	0.138403	64	2.056604	0.198717	53
	80	3.411765	0.295895	34	1.765625	0.15865	64	2.45283	0.214777	53
	100	4.029412	0.320108	34	1.875	0.18025	64	2.811321	0.208764	53
	120	4.441176	0.330265	34	1.953125	0.171695	64	3.018868	0.207778	53
	140	4.617647	0.304496	34	2	0.165172	64	2.981132	0.206024	53
D10	160	4.882353	0.320885	34	1.935484	0.135796	62	2.923077	0.19193	52
	180	4.941176	0.24597	34	1.983607	0.137288	61	2.692308	0.17911	52
	200	4.647059	0.256406	34	2.033333	0.162895	60	2.680851	0.199691	47
	220	4.441176	0.319288	34	1.864407	0.141912	59	2.6	0.180907	45
	240	4.151515	0.31116	33	1.833333	0.148954	54	2.142857	0.178949	42
	260	3.96875	0.346307	32	1.693878	0.152095	49	1.702703	0.159131	37
	280	3.741935	0.30398	31	1.666667	0.182574	36	1.518519	0.123694	27
	300	3.483871	0.34037	31	1.5	0.140859	28	1.333333	0.125988	15
	320	3	0.290957	28	1.619048	0.212426	21	1.25	0.130558	12
	20	1.235294	0.103923	34	1.107143	0.048874	56	1.140625	0.049135	64
	40	1.676471	0.182675	34	1.232143	0.072035	56	1.40625	0.110731	64
	60	2.558824	0.283822	34	1.321429	0.10227	56	1.609375	0.150425	64
	80	3.411765	0.295895	34	1.482143	0.111049	56	1.9375	0.186173	64

	100	4.029412	0.320108	34	1.517857	0.119499	56	2.375	0.224382	64
	120	4.441176	0.330265	34	1.571429	0.13924	56	2.703125	0.258467	64
	140	4.617647	0.304496	34	1.571429	0.136888	56	2.703125	0.246684	64
	160	4.882353	0.320885	34	1.518519	0.134119	54	2.733333	0.253636	60
**D14**	180	4.941176	0.24597	34	1.403846	0.087962	52	2.75	0.313392	56
	200	4.647059	0.256406	34	1.361702	0.093377	47	2.622642	0.27895	53
	220	4.441176	0.319288	34	1.4	0.106217	40	2.3125	0.261536	48
	240	4.151515	0.31116	33	1.361111	0.106553	36	2.175	0.220395	40
	260	3.96875	0.346307	32	1.333333	0.112367	33	2	0.196748	31
	280	3.741935	0.30398	31	1.357143	0.11745	28	1.96	0.241385	25
	300	3.483871	0.34037	31	1.227273	0.091449	22	1.55	0.169752	20
	320	3	0.290957	28	1.294118	0.113911	17	1.833333	0.270615	12
	20	1.235294	0.103923	34	1.148148	0.115762	27	1.268041	0.061334	97
	40	1.676471	0.182675	34	1.222222	0.134327	27	1.773196	0.106193	97
	60	2.558824	0.283822	34	1.481481	0.171887	27	2.268041	0.132316	97
	80	3.411765	0.295895	34	1.740741	0.210769	27	2.731959	0.159544	97
	100	4.029412	0.320108	34	1.814815	0.232888	27	3.061856	0.159745	97
	120	4.441176	0.330265	34	2.148148	0.290905	27	3.268041	0.16934	97
	140	4.617647	0.304496	34	2.444444	0.308167	27	3.185567	0.156702	97
	160	4.882353	0.320885	34	2.185185	0.26169	27	3.041237	0.146492	97
**D18**	180	4.941176	0.24597	34	2.111111	0.257831	27	2.793814	0.132659	97
	200	4.647059	0.256406	34	2.208333	0.288544	24	2.542553	0.141619	94
	220	4.441176	0.319288	34	1.875	0.227721	24	2.460674	0.14298	89
	240	4.151515	0.31116	33	1.954545	0.241543	22	2.209877	0.132306	81
	260	3.96875	0.346307	32	1.7	0.193309	20	2.04054	0.120838	74
	280	3.741935	0.30398	31	1.588235	0.211069	17	1.873016	0.116468	63
	300	3.483871	0.34037	31	1.375	0.154785	16	1.648148	0.102918	54
	320	3	0.290957	28	1.4	0.163299	10	1.418605	0.095482	43
	340	2.48	0.295071	25	1.333333	0.210819	6	1.37037	0.108711	27
	360	2.315789	0.342443	19	1.2	0.2	5	1.294118	0.113911	17
	20	1.235294	0.103923	34	1.144737	0.044737	76	1.16	0.094516	25
	40	1.676471	0.182675	34	1.539474	0.10993	76	1.36	0.14	25
	60	2.558824	0.283822	34	1.776316	0.139672	76	1.88	0.194251	25
	80	3.411765	0.295895	34	2.092105	0.169807	76	2.52	0.258972	25
	100	4.029412	0.320108	34	2.381579	0.198209	76	3.08	0.299555	25
	120	4.441176	0.330265	34	2.552632	0.195919	76	3.52	0.332265	25
	140	4.617647	0.304496	34	2.697368	0.195583	76	4.12	0.397157	25
	160	4.882353	0.320885	34	2.644737	0.194399	76	3.96	0.380701	25
**D21**	180	4.941176	0.24597	34	2.710526	0.201587	76	4.32	0.36842	25
	200	4.647059	0.256406	34	2.565789	0.176054	76	3.88	0.384361	25
	220	4.441176	0.319288	34	2.394737	0.164454	76	3.52	0.337244	25
	240	4.151515	0.31116	33	2.4	0.151865	75	3.44	0.327007	25
	260	3.96875	0.346307	32	2.27027	0.112685	74	3.04	0.297097	25
	280	3.741935	0.30398	31	2.271429	0.125309	70	2.8	0.310913	25
	300	3.483871	0.34037	31	2.045455	0.087524	66	2.583333	0.329232	24
	320	3	0.290957	28	1.87931	0.095506	58	2.380952	0.348303	21

The intersections of the dendrites of neurons in CA1

**Basal side**		**Sham**			**SNL**			**WTD**		
		**Mean**	**SEM**	**N**	**Mean**	**SEM**	**N**	**Mean**	**SEM**	**N**
	20	2.810811	0.200741	37	2.2375	0.116982	80	2.384615	0.174017	52
	40	4.459459	0.221226	37	4.2875	0.142529	80	4.442307	0.185063	52
	60	5.459459	0.283677	37	5.1125	0.145604	80	5.25	0.179817	52
	80	6.162162	0.298931	37	5.7625	0.158358	80	5.980769	0.193192	52
	100	5.972973	0.338887	37	6.075	0.160867	80	6.192307	0.184298	52
**D3**	120	6.142857	0.330883	35	6.05	0.162944	80	6.288462	0.198669	52
	140	5.914286	0.430695	35	5.7	0.180014	80	6	0.231397	52
	160	5.40625	0.401257	32	5.125	0.206979	80	5.254902	0.285387	51

	180	4.344828	0.382099	29	4.464789	0.212138	71	4.659575	0.334947	47
	200	3.321429	0.40517	28	3.245614	0.248152	57	4.142857	0.359977	42
	220	2.666667	0.374295	24	2.095238	0.189165	42	3.657143	0.354901	35
	240	1.736842	0.294612	19	1.655172	0.180829	29	3.172414	0.351173	29
	20	2.810811	0.200741	37	2.411765	0.27196	17	2.362069	0.131762	58
	40	4.459459	0.221226	37	3.705882	0.306363	17	4.172414	0.174159	58
	60	5.459459	0.283677	37	4.764706	0.278248	17	5.206897	0.230414	58
	80	6.162162	0.298931	37	5.352941	0.342366	17	5.724138	0.199505	58
	100	5.972973	0.338887	37	5.470588	0.374928	17	5.844828	0.222456	58
**D7**	120	6.142857	0.330883	35	5.294117	0.483285	17	5.965517	0.24958	58
	140	5.914286	0.430695	35	5.235294	0.525311	17	5.482759	0.28919	58
	160	5.40625	0.401257	32	4.705883	0.512812	17	4.431035	0.267307	58
	180	4.344828	0.382099	29	4	0.5	16	3.58	0.268161	50
	200	3.321429	0.40517	28	3.142857	0.49009	14	3	0.294254	41
	220	2.666667	0.374295	24	1.916667	0.228908	12	2.34375	0.299897	32
	240	1.736842	0.294612	19	1.25	0.163663	8	1.590909	0.234097	22
	20	2.810811	0.200741	37	2.529412	0.212091	34	2.48718	0.175688	39
	40	4.459459	0.221226	37	3.941176	0.223173	34	3.794872	0.152264	39
	60	5.459459	0.283677	37	4.911765	0.199677	34	5.128205	0.21477	39
	80	6.162162	0.298931	37	5.382353	0.206898	34	5.692307	0.241324	39
	100	5.972973	0.338887	37	5.5	0.236017	34	5.769231	0.199397	39
**D10**	120	6.142857	0.330883	35	5.5	0.277659	34	5.666667	0.218365	39
	140	5.914286	0.430695	35	4.911765	0.251086	34	5.105263	0.246712	38
	160	5.40625	0.401257	32	4.264706	0.29647	34	4.105263	0.321778	38
	180	4.344828	0.382099	29	3.212121	0.252853	33	3.242424	0.30473	33
	200	3.321429	0.40517	28	2.433333	0.247671	30	2.538461	0.314915	26
	220	2.666667	0.374295	24	1.6875	0.236621	16	2	0.284398	17
	20	2.810811	0.200741	37	2.48718	0.213801	39	2.588235	0.145996	51
	40	4.459459	0.221226	37	4.025641	0.221902	39	4.392157	0.215258	51
	60	5.459459	0.283677	37	4.641026	0.233749	39	5.098039	0.227958	51
	80	6.162162	0.298931	37	5.051282	0.254378	39	5.666667	0.237085	51
	100	5.972973	0.338887	37	5.102564	0.291107	39	5.803922	0.215151	51
**D14**	120	6.142857	0.330883	35	4.81579	0.321924	38	5.705883	0.25095	51
	140	5.914286	0.430695	35	4.222222	0.293071	36	5.078432	0.262482	51
	160	5.40625	0.401257	32	3.657143	0.28972	35	4.265306	0.273202	49
	180	4.344828	0.382099	29	3.090909	0.343676	33	3.487805	0.306678	41
	200	3.321429	0.40517	28	2.346154	0.332553	26	2.821429	0.37109	28
	220	2.666667	0.374295	24	1.727273	0.199369	22	2.227273	0.353901	22
	240	1.736842	0.294612	19	1.714286	0.244243	14	2	0.467099	11
	20	2.810811	0.200741	37	1.952381	0.200905	21	2.59322	0.141212	59
	40	4.459459	0.221226	37	3.571429	0.263416	21	4	0.145053	59
	60	5.459459	0.283677	37	4.238095	0.247894	21	4.79661	0.161777	59
	80	6.162162	0.298931	37	4.523809	0.289655	21	5.118644	0.212256	59
	100	5.972973	0.338887	37	4.761905	0.371001	21	5.423729	0.20256	59
**D18**	120	6.142857	0.330883	35	4.380952	0.361717	21	5.508474	0.228548	59
	140	5.914286	0.430695	35	3.857143	0.432522	21	4.830509	0.250251	59
	160	5.40625	0.401257	32	3.421053	0.369256	19	3.864407	0.256371	59
	180	4.344828	0.382099	29	2.944444	0.365794	18	2.888889	0.2418	54
	200	3.321429	0.40517	28	2.357143	0.341412	14	2.255814	0.188522	43
	220	2.666667	0.374295	24	1.888889	0.388889	9	1.518519	0.144908	27
	240	1.736842	0.294612	19	1.285714	0.184428	7	1.3	0.152753	10
	20	2.810811	0.200741	37	2.390625	0.119143	64	2.636364	0.242154	22
	40	4.459459	0.221226	37	3.53125	0.129806	64	4.181818	0.224441	22
	60	5.459459	0.283677	37	3.90625	0.143854	64	5.181818	0.352089	22
	80	6.162162	0.298931	37	4.140625	0.150836	64	5.727273	0.33017	22
	100	5.972973	0.338887	37	4.1875	0.164984	64	5.863636	0.356118	22
**D21**	120	6.142857	0.330883	35	4.126984	0.17888	63	5.954545	0.325518	22
	140	5.914286	0.430695	35	3.66129	0.186741	62	5.863636	0.373908	22

160	5.40625	0.401257	32	3.070175	0.198458	57	5.045455	0.443702	22
180	4.344828	0.382099	29	2.28	0.189608	50	4.045455	0.453353	22
200	3.321429	0.40517	28	1.736842	0.154055	38	3.117647	0.491713	17
220	2.666667	0.374295	24	1.380952	0.12866	21	2.25	0.391675	12

Supplementary data sheet 1.5

The intersections of the dendrites of neurons in CA3

Apical side		Sham			SNL			WTD		
		**Mean**	**SEM**	**N**	**Mean**	**SEM**	**N**	**Mean**	**SEM**	**N**
**D3**	20	2.02432	0.192409	50	1.607143	0.194768	28	1.842105	0.2785	19
	40	2.69364	0.244872	50	2.285714	0.256436	28	2.631579	0.376274	19
	60	3.65608	0.250972	50	2.714286	0.2763	28	3.894737	0.431878	19
	80	4.38776	0.260631	50	3.035714	0.297619	28	4.526316	0.392685	19
	100	4.72622	0.292698	50	3.357143	0.287363	28	4.578948	0.377091	19
	120	4.490816	0.311749	49	3.107143	0.27381	28	4.166667	0.519238	18
	140	4.136191	0.304942	47	2.923077	0.259722	26	3.9375	0.469652	16
	160	3.19775	0.249805	44	2.636364	0.275598	22	3.153846	0.450728	13
**D7**	20	1.235294	0.103923	34	1.217391	0.087939	23	1.441558	0.087678	77
	40	2.69364	0.244872	50	1.65625	0.171623	32	1.792453	0.115423	53
	60	3.65608	0.250972	50	1.96875	0.203222	32	2	0.126353	53
	80	4.38776	0.260631	50	2.225806	0.261138	31	2.098039	0.135052	51
	100	4.72622	0.292698	50	2.483871	0.265876	31	2.122449	0.156037	49
	120	4.490816	0.311749	49	2.448276	0.269949	29	2.041667	0.157435	48
	140	4.136191	0.304942	47	2.392857	0.283307	28	1.9	0.163299	40
	160	3.19775	0.249805	44	2.16	0.280951	25	1.75	0.173902	32
	180	2.908029	0.251895	34	2.210526	0.346907	19	1.45	0.135239	20
**D10**	20	2.02432	0.192409	50	1.333333	0.09759	36	1.585366	0.130551	41
	40	2.69364	0.244872	50	1.777778	0.178668	36	1.707317	0.168363	41
	60	3.65608	0.250972	50	1.944444	0.182333	36	1.780488	0.176387	41
	80	4.38776	0.260631	50	2.090909	0.210355	33	1.783784	0.182307	37
	100	4.72622	0.292698	50	2.333333	0.221455	30	1.75	0.19572	32
	120	4.490816	0.311749	49	2.115385	0.202499	26	1.875	0.183687	24
	140	4.136191	0.304942	47	2	0.208514	23	1.75	0.175844	20
	160	3.19775	0.249805	44	1.95	0.245753	20	1.733333	0.228174	15
	180	2.908029	0.251895	34	1.785714	0.238553	14	1.555556	0.293972	9
**D14**	20	2.02432	0.192409	50	1.230769	0.159882	26	1.136364	0.099685	22
	40	2.69364	0.244872	50	1.269231	0.162269	26	1.5	0.205445	22
	60	3.65608	0.250972	50	1.423077	0.148563	26	2.090909	0.359829	22
	80	4.38776	0.260631	50	1.615385	0.147564	26	2.136364	0.343747	22
	100	4.72622	0.292698	50	1.434783	0.138114	23	2.5	0.387298	20
	120	4.490816	0.311749	49	1.571429	0.162882	21	2.315789	0.375045	19
	140	4.136191	0.304942	47	1.5	0.166667	18	2.111111	0.341937	18
	160	3.19775	0.249805	44	1.538462	0.183114	13	2.357143	0.414134	14
	180	2.908029	0.251895	34	1.555556	0.242161	9	2.3	0.448454	10
**D18**	20	2.02432	0.192409	50	1.34375	0.096401	32	1.5625	0.18186	16
	40	2.69364	0.244872	50	1.625	0.153914	32	2.1875	0.163777	16
	60	3.65608	0.250972	50	2	0.1905	32	2.5	0.204124	16
	80	4.38776	0.260631	50	2.125	0.209406	32	2.8125	0.291815	16
	100	4.72622	0.292698	50	2.21875	0.223311	32	2.875	0.286865	16
	120	4.490816	0.311749	49	2.322581	0.224265	31	2.25	0.266145	16
	140	4.136191	0.304942	47	2.285714	0.234899	28	2.363636	0.278722	11
	160	3.19775	0.249805	44	1.962963	0.216693	27	1.909091	0.414609	11
	180	2.908029	0.251895	34	1.703704	0.175532	27	1.9	0.433333	10
**D21**	20	2.024	0.1924	50	1.511	0.1217	43	1.692	0.1347	42
	40	2.694	0.2449	50	2.051	0.1372	43	2.435	0.2436	42
	60	3.656	0.251	50	2.303	0.1584	42	3.537	0.2672	42
	80	4.388	0.2606	50	2.224	0.1721	41	4.283	0.3031	42
	100	4.726	0.2927	50	2.151	0.1639	33	4.539	0.3041	42
	120	4.491	0.3117	49	1.994	0.1651	29	4.559	0.2855	42
	140	4.136	0.3049	47	1.792	0.1664	23	4.098	0.293	42
	160	3.198	0.2498	44	1.315	0.1508	16	3.399	0.2551	40
	180	2.908	0.2519	34	1.297	0.1521	9	2.553	0.2433	34

The intersections of the dendrites of neurons in CA3

Basal side		Sham			SNL			WTD		
		Mean	SEM	N	Mean	SEM	N	Mean	SEM	N
	20	2.352941	0.192934	34	1.8	0.119829	40	2.090909	0.205804	33
	40	4.117647	0.23034	34	3.3	0.196769	40	3.515152	0.272201	33
	60	4.764706	0.267219	34	4.325	0.23585	40	4.333333	0.256235	33
	80	4.823529	0.284698	34	4.9	0.231495	40	4.939394	0.238125	33
	100	4.911765	0.284375	34	5.2	0.245994	40	5.3125	0.251758	32
**D3**	120	4.676471	0.309449	34	4.975	0.254416	40	5.28125	0.270284	32
	140	4.15625	0.33029	32	4.375	0.303162	40	4.90625	0.340065	32
	160	3.033333	0.255889	30	3.815789	0.308383	38	4.9	0.353472	30
	180	2.481482	0.279246	27	2.857143	0.323175	35	4.517241	0.373443	29
	200	2.25	0.323427	20	1.96	0.261279	25	3.892857	0.361286	28
	220	2	0.308607	15	1.722222	0.350588	18	3.48	0.379122	25
	20	2.581316	0.167384	38	2.461539	0.201767	26	2	0.158495	46
	40	4.303158	0.291267	38	3.346154	0.282529	26	3.021739	0.209645	46
	60	5.658684	0.297447	38	3.653846	0.327895	26	3.434783	0.231884	46
**D7**	80	5.949053	0.357832	38	3.884615	0.377551	26	3.652174	0.281846	46
	100	5.372189	0.364276	37	3.884615	0.423636	26	3.478261	0.284442	46
	120	4.587371	0.36405	35	3.333333	0.424207	24	3.071429	0.262643	42
	140	3.455194	0.355923	31	3	0.432591	19	2.769231	0.236545	39
	20	2.352941	0.192934	34	1.769231	0.230769	26	2.15625	0.174535	32
	40	4.117647	0.23034	34	2.961539	0.274455	26	3.09375	0.221612	32
	60	4.764706	0.267219	34	3.346154	0.308557	26	3.375	0.24899	32
	80	4.823529	0.284698	34	3.692308	0.322344	26	3.59375	0.307367	32
**D10**	100	4.911765	0.284375	34	3.192308	0.350908	26	3.466667	0.324244	30
	120	4.676471	0.309449	34	3.173913	0.359249	23	3.185185	0.315944	27
	140	4.15625	0.33029	32	2.681818	0.402301	22	2.541667	0.275702	24
	160	3.033333	0.255889	30	2.333333	0.392388	21	2	0.297044	17
	180	2.481482	0.279246	27	2.105263	0.425054	19	1.846154	0.355293	13
	20	2.578947	0.167109	38	2.172414	0.227803	29	2.1	0.228266	20
	40	4.302631	0.291132	38	3.62069	0.299482	29	3.4	0.265568	20
	60	5.660526	0.297537	38	3.724138	0.232234	29	3.75	0.25	20
**D14**	80	5.947369	0.358076	38	3.965517	0.269477	29	4.1	0.306937	20
	100	5.37027	0.363435	37	3.862069	0.373784	29	4.105263	0.381554	19
	120	4.588572	0.363428	35	3.444444	0.326133	27	4.263158	0.457824	19
	140	3.451613	0.35605	31	3.2	0.294392	25	3.526316	0.491854	19
	20	2.578947	0.167109	38	1.827586	0.172414	29	2.571429	0.234738	21
	40	4.302631	0.291132	38	3.275862	0.215739	29	3.761905	0.33739	21
	60	5.660526	0.297537	38	3.62069	0.218672	29	4	0.351866	21
**D18**	80	5.947369	0.358076	38	4	0.257881	29	4.238095	0.357936	21
	100	5.37027	0.363435	37	4.137931	0.300755	29	4.285714	0.39123	21
	120	4.588572	0.363428	35	4.107143	0.326677	28	3.9	0.390007	20
	140	3.451613	0.35605	31	4.148148	0.356877	27	3.722222	0.36876	18
	20	2.581	0.1674	38	1.884	0.1062	43	3.293	0.2424	41
	40	4.303	0.2913	38	2.213	0.135	42	5.121	0.2954	41
	60	5.659	0.2974	38	2.454	0.1883	35	6.313	0.2839	41
**D21**	80	5.949	0.3578	38	2.633	0.2178	27	6.746	0.3814	41
	100	5.372	0.3643	37	2.67	0.2263	23	5.864	0.428	40
	120	4.587	0.3641	35	2.134	0.2948	19	4.697	0.4609	38
	140	3.455	0.3559	31	1.961	0.3009	12	3.562	0.4552	34

Supplementary data sheet 2.1

The number of neurons double stainied with TMEM119 and TNFα in CA1			The number of neurons double stainied with TMEM119 and TNFα in CA3		
Sham	SNL	WTD	Sham	SNL	WTD
1.9	8.4	2.9	1.8	16.2	1.1
1.8	8.8	2.4	1.7	8.8	2.8
2.2	10	3	1.4	10.1	2

Area of the cell body of microglia in CA1			Area of the cell body of microglia in CA3		
Sham	SNL	WTD	Sham	SNL	WTD
1476	1677	3798	2334	3346	1394
1664	3890	3738	1440	2472	2068
1076	1770	2970	1552	2873	1244
708	3598	1594	2000	3338	1044
1564	4058	2590	2214	5208	956
1530	1840	2516	1162	5148	1648
970	1734	3180	2118	6746	1492
882	3304	2712	1888	2436	842
720	2874	1480	1204	5310	1248
1158	3486	1562	1282	5092	1302
474	3476	3804	1454	4480	976
1446	2754	3370	1472	3676	1592
1402	2972	4394	1070	4460	1424
856	3164	2422	1536	4844	1042
2198	1238	3918	1248	4954	1610
2010	3012	3432	2410	3600	1080
1434	4010	3704	1066	6330	1624
1226	3435	2422	1206	2348	1058
1680	3226	1794	996	2626	1600
1308	4930	2867	1642	3166	1414
988	2892	3618	1386	5460	986
1138	4316	1986	1204	4248	1556
924	2896	2198	1276	3040	1112
1268	3524	5646	1322	3682	1044
1050	5544	3644	1438	3901	1634
1370	5182	2518	2152	7372	1316
768	4600	3156	2124	3466	1226
1632	4080	3692	1552	2970	1564
4282	4830	3370	1404	3889	3659
1626	6466	2692	1504	4160	1334
1976	4680	3224	1426	4889	1828
2790	2856	1264	1860	5136	1910
1500	4404	1880	1962	5000	1570
1470	4042	2002	1998	5806	2016
1920	4550	3534	758	5862	1848
2122	2924	3340	1152	4164	1406
2092	4416	1564	806	5276	1744
2032	5184	1808	1226	5744	2646
1830	3742	4234	1044	5446	2632
1424	4140	2150	1322	4000	1610
1552	5146	2016	1160	3210	2202
1982	4794	1384	1268	4190	2270
2066	3180	2481	2406	5970	1238

1470	3946	1880	1260	7044	1326
1616	5128	5004	1144	4110	2352
1456	5056	3978	1414	4586	1122
	7406	5574	944	4834	1620
	6236	3430	864	4578	1262
	3618	5300	1550	3974	1664
	7206	3806	1914	4320	2094
	4122	2822	1180	4756	1824
	4038	3112	1554	4970	1864
	6203	3000	1134	5478	1474
	3928	3750	2137	7266	2068
	3810	1542	1856	6040	1798
	4801	2412	1324	3860	1542
		1846	2212	3542	1436
		3322	1200	4312	1540
		2118	1206	4728	2382
		2646	1440	3516	1106
		1436	1828	4388	1576
		2140	1228	5456	1050
		2962	1658	5932	2076
		2004	1694	4442	1436
		2202	1094	5603	1172
		1972	2022	5416	1938
		3468	1544	4210	1704
		1900	1889	4880	1152
		2993	1710	2896	2008
		3260	1180	5238	1384
		4720	1222	3934	934
		2558	1344	5066	1550
			1582	7458	
			1542	4362	
			1082	4056	
			1090	4414	
			1734	4030	
				3568	

Supplementary data sheet 2.2

Spine numbers in primary dendrites (10μm)								Numbers of stubby spines in primary dendrites (10μm)							
Group1	Group2	Group3	Group4	Group5	Group6	Group7	Group8	Group1	Group2	Group3	Group4	Group5	Group6	Group7	Group8
5.1	3.4	3.6	3.9	1.4	2.6	7.5	7.2	2.8	2.9	1.8	3.9	1.4	0	5	3.6
5.6	9.1	2.8	1	2.6	2.1	6.1	4.4	3.3	7.1	1.8	0	1.1	1.6	4.1	0.9
2.5	6.2	2.2	2.7	2.7	3	7.2	5.5	2.5	2.9	1.3	0.9	1.3	2	3.6	3.7
3.2	3.4	0.5	3.4	2.7	2.8	5.8	5.5	3.2	0.5	0.5	1.5	0.4	0.5	1.5	4
3	7	1.7	1.9	2.4	1	4.3	5	1.3	5	1.3	0	0.5	1	2.2	3
1.8	5	0.8	1.5	2	1.6	4.2	5.8	0.9	2	0.8	1.5	1.4	1.6	2.3	1.8
4.1	5.4	1.1	2.6	1.9	2	6.7	6.3	1.8	1.5	0.5	1	1.9	0.8	2.9	3.1
3.7	6.2	3.2	1	1.1	2.6	4.7	4.3	0.8	2.9	2	0	1.8	1.6	2.3	3.4
2.9	3.7	2.4	2.3	1.7	2.8	5.8	2.5	1.9	2.6	1.7	0.5	0.6	2.8	2.9	0.5
1.9	6.8	1.9	1.4	2.1	1.3	5.6	9	1.4	1.5	1.7	0.5	2.1	0	3.8	6
1.3	5.9	2.1	0.9	1.6	3.8	5.9	3.9	1.8	1.4	1.1	0.5	1.6	1.9	1.8	1.5
2.7	5.6	3.3	2	1.2	5.8	5.4	4.2	0.7	2.6	1.5	1.2	1.2	2.1	2.2	2.8
3	5.6	1.3	2.2	0.9	5.2	5.1	4.8	1.1	3.5	0.8	1.6	0.8	4.1	3	2.1
2.3	6.9	2.3	5.3	1.5	2.3	2.9	4.7	1.9	1.6	0.5	1.6	2.1	1.4	1.2	2.6
0.9	6.5	1.9	2	2.4	2.4	6.7	5.6	0.7	2.3	1.1	2	0.6	1.4	3.9	2.1
1	6.1	3	0.9	2	1.8	5.6	4.5	1.3	3	1.2	0.9	2.2	1.3	2.8	2.7
3.4	2.1	2.7	1	1.9	2.7	5.9	4.4	2	1.1	1.4	1	1.9	2.7	2.3	2
2	8.4	2.9	3	1.4	4.4	4.8	8.3	1.6	2.1	1	1.7	0.7	3.3	1.3	4.1
1.3	5.1	1.6	4.8	1	2.5	4.3	4.2	0.5	0.9	1.3	2.7	1.8	0.5	2.4	2.6
2.4	5.4	1.4	6.1	1.6	1.6	4.9	4.3	0.8	1.4	0.9	2.8	1.4	1.1	1	1.4
2.2	7.6		4	1.2	4	3.7	7.6	1.1	3.1		1.5	2.4	1.3	1.9	4.6
2.2	10.9		2.3	0.8	4.9	4.3	4.9	2.2	5.4		1.4	1.6	3.3	1.9	1.8
4.8	5.6		1.4	0.7	2.1	5.7	6.8	3.4	2.2		0.9	1.9	0.5	3.8	2.3
4.5	5.2		1.5		3.7	5.9	6.6	1.8	2.9		0.5		0	3.4	2.3
5.6	8.3		1.4		0.8	6.9	4.9	5.6	1.4		0.5		0.4	4.9	1.3
2.5	9.2		1.8		3.3	5	6.5	1.2	3.1		0.9		1.9	3.5	1.4
2.7	3.5		1.5		3.2	5.8	7.3	1.3	2		0.5		2.3	3.9	4.2
1.4	10.2		2.3		5.5	3.9	6.1	0	3.8		1.4		3	1.7	1.5
3.2	7.3				2.5	4.3	5.5	2.3	1.9				1.7	2.2	1.8
1.3	4.6				2.9	5.2	7.4	0.9	0.9				2.1	1.9	1.9
3	3.2				4.1	5.3	6.4	3	1.1				2.5	3.4	5
	7.8					5.6	7		4.9					4.1	3.3
	5.4					3.4	9.2		2.7					2.1	4.6
	9.6					8.2	7.1		7					3.5	6.3
	6.5					6.2	10.1		3.5					2.6	4.2
	11.7					5.5	7.8		5.9					3.5	6.9
	14.5					4.6	8.3		10.5					1.4	5
	9.3					3.8	8		5.4					0.4	4.9
	9.9						7.3		4.4						5.2
	9.2						6.9		3.2						4.6
	8.1						9.4		3.6						4.2
	6.2						7.9		3.3						6.1
							6.6								5.4
							11.3								4.4
							9.9								7.2
															6.1

Numbers of thin spines in primary dendrites (10μm)

Group1	Group2	Group3	Group4	Group5	Group6	Group7	Group8
1.8	0.5	0.9	0.6	0	2.2	1.3	2.3
2.3	1	0	0.5	0.5	0.5	1	1.3
0	2.9	0.4	0.4	0.4	0	2.1	1.4
0	2	0	1	0.9	1.9	1.7	1
1.7	1.5	1.1	0.5	0.9	0	1.3	2.7
0.9	2	1.7	0	1.6	0	1.9	2.1
1.4	2	1	0.5	1.4	1.2	1.9	2
1.5	2.4	1.2	0	0.4	0	0.9	1
0.5	0.5	0.4	0.5	0	0	1	1.5
1.3	3.4	0.7	0.5	2.1	1.3	0.9	1.6
1	2.3	0.5	0	0.9	1	2.7	1
1.4	1.7	1.5	0	1.5	2.1	2.7	0.9
2.1	1.5	1	0.5	1.8	1.2	1.3	1.5
1.9	4.2	0.8	2.1	0.4	0.9	1.8	1.8
1.6	2.3	0.8	0	0.9	0.5	1.2	1.9
1	1.5	1.3	0.2	1.1	0.4	0.9	1.5
0.9	1.1	1.4	0	1.5	0	2.3	1.3
0.8	3.1	2	1.3	0.6	0.5	1.7	2.3
1.4	2.3	1.2	0.5	1.3	2	1	0.9
0.4	2.3	1	1.9	0	0.5	2.4	2.3
0.5	1.9		2	2.2	0.9	1.4	1
1.3	1.8		0	1.6	1.6	1.4	1
1	2.2		0.5	0.9	1.1	0.5	2.8
2.1	1.2		0.5		1.6	1.7	1
1.9	4.2		0.9		0.4	1.6	1.2
1.3	3.6		0.4		1.4	1	2.3
1.9	0.5		1		0.9	1	2.1
1.9	4.3		0.5		2.5	1.3	1.9
0.9	3.9				0.8	1.3	2
1	2.3				0.8	1.9	2.1
0.9	1.1				1	1.4	1.8
2.7	1.5					1	2.2
0	1.8					1.4	1.5
0.5	1					2.3	1.9
1.7	2					2.2	1.5
0	3.9					1	2
1.2	2.5					1.9	2.2
0.9	2.9					2.1	1.7
1.4	3.9						3
0.9	3.2						2.7
0.4	2.7						3.3
0	1.9						2.9
							2.2
							4.1
							3.9

Numbers of mushroom spines in primary dendrites (10μm)

Group1	Group2	Group3	Group4	Group5	Group6	Group7	Group8
0.5	1	0.9	0	0.4	0	1.3	1.4
0	0.5	0.9	0.5	0	1.1	1	2.2
0	1	0.4	1.3	1	0.9	1.5	0.5
0	0.5	0	1	0.5	0.7	0.5	1
0	1	0.4	1.4	0	0.9	0.9	1
0	2	0.7	0	0	0	0	1.3
0.9	1	0	1	0	1.4	1.9	1
0.4	0.5	0.6	1	1	0	1.4	1
0.5	1.9	0.5	1.4	0	0.9	1.9	1
0.5	2.3	0	0.5	0	1	0.9	1.5
0.7	1.3	0	0.5	1	0.6	1.4	1.9
0.8	0.5	0.7	0.8	1.6	0.5	0.4	0.9
0.2	1.1	0.5	0	0	0.2	0.9	1.1
0.6	1.9	0.9	1.6	0	1.2	1.7	1
0	1.5	0.3	0	0.5	1.5	1.6	1
0.3	3.1	0.4	0	0	0.7	1.9	0.9
0.5	1.9	0	0	0	0.5	1.4	1
0	1.8	0.2	0	0.5	1.4	1.7	2.3
0.7	2.5	0.1	1.6	0	0	1	1
0	3.6	0.5	1.4	0	0.3	1.5	1
0.3	1.3		0.5	1.8	0.8	0.5	1.5
0.4	1.2		0.9	0	0.3	1	1.8
1	2.8		0	0.5	1	1.4	2.3
0	2.6		0.5	2.1		0.8	1.4
0	1		0	0		0.4	3.1
0	2.1		0.4	0		0.5	2.8
0.4	1.5		0	0		1	2.1
0	1.4		0.5	0		0.9	3.6
0	1.1			0		0.9	0.9
0	1.5			0		1.4	1.5
0	0.9			0.5		0.5	3.1
	1.5					0.5	2.7
	1					0.9	2.4
	2					1.7	3.3
	1.5					1.3	
	1					1	
	1.7					1.4	
	2.7					1.3	
	1.8						
	2.4						

Spine numbers in secondary dendrites (10μm)

Group1	Group2	Group3	Group4	Group5	Group6	Group7	Group8
1.8	5.2	2.3	0	1.3	2.3	5	3.7
1	4.1	0.4	1.8	1.5	1.3	7.9	2.2
2.2	5.2	0.5	2.7	1.5	2.3	5.9	6.4
1.8	4.4	2.2	1.7	1.2	2.6	5.5	4.9
2.4	6.7	1.1	1.7	3.2	1.6	4.9	3.7
1.9	6.4	0.8	2.7	0.9	2.6	9.7	4.9
2.5	3.4	1.4	0.5	2.9	4.3	5	3.5
1.4	3	0.1	0.5	1.5	2.8	3.5	5.6
1.3	6.4	0.9	1.5	1.9	4.4	5.6	3.3
2.9	4.6	1.6	1	1.1	3	6	3.7
2.3	4.5	1.4	3.3	0.7	1.9	3.1	5.7
1.5	4.1	0.9	1.4	2.1	2.9	3.6	3.8
1.9	3.1	1.1	2.3	0.7	2.8	4.5	4.8
0.8	6.6	0.6	1.3	0.8	2.7	4.6	5.1
1.3	4.3	0.8	3	0.8	1.4	6.6	2.9
3.7	3.6	1.5	0.9	1.1	4.7	3.8	4.3
2.4	4	2	0.5	1	5.9	3.7	4.9
1.1	5.3	1.4	1	1.4	3.9	2.8	3.8
1.6	6.4	1.3	0.5	1.5	2.3	3.3	5
0.9	3.9	1.8	0.9	1.7	1.5	2.9	2.8
1.7	5.1	2.1	2.6	0.9	2.1	5.6	4
1.4	2.4	0.9	1.7	1	1.8	2.5	5
3.8	5.4	2.4	2.4	1.4	3.1	3.8	4.2
3	5.1		0.5	1.2	1.9	3.7	4.1
1.4	2.6			2	3.3	5.7	4.1
3.2	5.6				5	6.3	4.5
3.2	5.3				0.9	6.4	4
1.4	3.9				2.5	4	3.9
1.8	5.9				1.4	1.8	4.2
2.8	3.3				3.2	3.4	2.6
2.3	4.3				2.4	2.7	5.3
2.9	5				2.6	3.2	5.8
1.4	3.7				3	4	4.2
1.8	6.7				4.4	4.6	3.3
	6.3				2.7	2.8	5.2
	2.3					3.1	5.4
	4.2					8.2	4.1
	5.6					3.4	3.9
	5.4					3.3	4.6
	4.5					3.7	4.3
	1.7					4.7	4.2
	5					4.9	
	6.3					5.6	
	4.3					3.7	
	6					2	

Numbers of stubby spines in secondary dendrites (10 μm)

Group1	Group2	Group3	Group4	Group5	Group6	Group7	Group8
1.3	2.1	1.8	0	1.3	1.4	2.7	1.8
0.5	0.9	0.4	0.9	1.5	0.4	5.1	0.4
1.3	2.4	0	2.7	1.2	1.9	3.2	1.7
1.4	2.5	0.9	0.4	0.8	1.5	2.6	0.4
0.5	2.2	1.6	0.8	1.8	1.6	2.5	1.8
0.5	4.6	1.3	2.3	0.4	1.6	2	1.8
2	1.7	0.6	0.5	1.6	2.7	3.1	0.9
1.4	2.3	0.9	0.5	0.7	1.9	2.1	2.2
1.3	2.6	1.5	1	0.5	3.4	1.5	2.1
1	1	1.9	0.5	1.1	1	2	2.5
1.4	2.7	1.1	2.4	1	1.9	4.5	2.8
1.5	2.7	0.7	0.5	0.7	2.9	1.4	1.3
1.9	1.3	0.6	0.9	1.1	2.3	1	2.6
0.4	1.8	1.5	0.4	0.6	1.8	1.1	1.4
0.9	1.6	1.1	1.5	0.9	0.5	1.7	2.9
1.6	2.7	0.4	0.5	1.5	2.4	1	2.4
1	0.7	0.5	0.5	0.4	3.8	2.6	1.3
1	3.1	1	0.5	1.2	1.3	1	1.4
1.2	2.1	1.3	0.5	0.6	1.4	2.1	2.9
0.7	1.7	0.8	0.4	0.6	1	2.3	1.8
1.3	2.8	0.6	1.7	1	1.6	1.9	1.8
0.6	1	1	0.8	1.3	0.9	1.9	0.9
1.9	2.5	1	1.4	0.7	1.3	1.7	0.8
2	1.7		0.5	0.7	1.9	2.5	1.6
1.4	1			0.5	2.2	0.9	1.8
3.2	2.5				1.1	1.1	1.8
2.3	4				0.9	2.3	0.9
0.9	1.3				0	3.2	0.9
0.9	2				0.9	3.5	1.4
2.3	2.5				1.6	2	1.7
1.8	1.3				2.4	2.4	1.8
2.4	3.6				1.5	0.5	1.9
0.9	1.4				1.5	5.3	1.4
1.8	2.2				3.5	1	0.5
	2.9				2.2	2.2	1.7
	1.9					1.4	1.2
	2.8					1.6	2.3
	3.7					1.5	0.5
	3.6					1.5	1.4
	3.3					2.1	0.9
	0.4					0.4	1.4
	3.3					1.5	3.6
	3.1					1.5	5.4
	1.3					0.8	4.4
	4.2					1.1	3.5

	5.7					4.1			1.9					1.4	
	5.3					3.5			2.4						
	5					2.4			1.4						
	3.5					2.3			1.8						
	6.1					2.4			4.1						
	7.4								4.6						
	4.3								2.7						
	4.3								2.4						
	4.6								2.8						
	3.8								1.6						
	3.3								0.9						

**Numbers of thin spines in secondary dendrites (10μm)**								**Numbers of mushroom spines in secondary dendrites (10μm)**							
**Group1**	**Group2**	**Group3**	**Group4**	**Group5**	**Group6**	**Group7**	**Group8**	**Group1**	**Group2**	**Group3**	**Group4**	**Group5**	**Group6**	**Group7**	**Group8**
0.4	1.6	0	0	0	0.5	0.9	1.8	0	1.6	0.5	0	0	0.5	0.9	4.4
0.5	2.7	0	0.4	0	0.4	0.5	1.3	0	2.5	0	0.4	0	0.4	2.5	3.4
0	1.6	0	0	0	0.5	1	2.1	0.9	1.2	0.5	0	0.4	0	0.8	2.5
0.5	1.5	0.9	0.9	0	1	2	2.7	0	1.5	0.4	0.4	0.4	0	0	1.8
0.9	2.7	1	0.4	0.9	0	1.1	0.9	0.9	1.8	0.7	0.4	0.5	0	2.2	2.9
1.4	0.9	0.7	0.5	0.4	1	2.3	1.8	0	0.9	0.4	0	0	0	2.4	1.3
0.5	1.3	0.7	0	0.4	1.6	0.9	1.3	0	2.3	0	0	0.8	0	1.8	1.3
0	0.8	0.4	0	0	0.9	0.4	2.6	0	2	0.5	0	0.7	0	1.3	3.9
0	2.1	0	0	0	0.5	1.5	0.8	0	1.7	0.7	0.5	0.6	0.5	2.7	0.9
1.9	2.1	0	0	0	1	2.5	0.9	0	1.5	0.3	0.5	0	1	2.5	1.2
0.5	0.9	1	0.5	0.7	0	0.4	2.1	0.5	0.9	0	0.5	0.9	0	0	1.9
0	0.9	0.6	0.9	0.5	0	1	0.9	0	1.5	0	0	0.3	0	1.4	1.3
0	1.3	0	0.9	0	0.5	1.5	0.9	0	1.4	0.5	0.5	0.5	0	3.3	2.8
0.4	1.8	0.4	0.4	0	0.9	1.5	0.9	0	3.1	0.4	0.4	0	0	2.2	1.8
0.4	1.9	0	1	1	0.9	1.5	1.8	0	0.8	0	0.5	0	0	0.8	1
0.8	0.4	0.6	0	0.7	1.2	1	1.4	1.2	2.4	0	0.5	0.4	1.2	3.8	1.2
0.5	2.7	0	0	0.5	1.7	0.5	0.8	0.6	0.7	0.8	0	0.4	0.4	4	0.5
1	1.8	0	0.5	0	1.7	0.6	0.5	0	3.4	0	0	0.7	0.9	0	1.8
0.7	1.7	0.8	0	0	0.9	1.7	0.4	0.3	2.6	0.9	0	0.6	0	3.3	2.7
0.8	1.7	0.4	0	0.6	0.5	1	1.4	0	0.4	0.6	0.4	0	0	2.7	0.8
0	2.3	1.1	0.4	0.4	0.5	1.5	2.5	0.7	3.1	0.4	0.4	0.7	0	4	1.2
0.4	0.5	0.6	0.4	0	0.4	0.5	1.2	0.4	1	0	0.4	0.3	0.4	1.4	1.8
1.9	2.5	0	0.5	0.3	1.9	1.1	0.5	1.9	0.4	0.5	0.5	0.5	0	2.5	1.8
0.5	1.7		0	0.4	0	0.5	0.9	0.5	1.7		0	0.4	0	4.2	2.6
0	0.5			0.8	1.1	1.4	0.4	0	1			0	0	0	0.9
0	2				0.6	1	2.2	0	1				3.4	1.8	0.9
0.9	0.9				0	0.5	1.9	0.9	2.7				0	1.3	1.9
0.5	2.2				2	2.3	0.9	0.5	4.5				0.5	0	2.7
0.5	2.5				0	2	0.9	0.5	1.5				0.5	1.7	2.4
0.5	2.8				1.1	0.4	1.5	0.5	2.4				0.5	0	0.9
0.5	2.6				0	2.6	1.9	0.5	0.4				0	1.1	0.9
0.5	0.9				1	2.4	1.9	0.5	2.9				0	1.1	2.2
0.5	1.9				1	1.9	1.3	0.5	2.5				0.5	0	1.7
0	1.8				0.4	1.1	2.5	0	2.7				0.4	0	0.9

2.9	0	0.9	0.9	3.5	0.4	0.7	2.4
0.5		1.6	1	4.2		1.4	1.8
0.9		1.5	1.4	1.4		0	1.3
0.9		2.6	2.1	0.9		3.8	5.7
0.8		0.5	0.9	0.9		3.3	5
0.8		0.8	4.4	1.7		4	
1.2		1	3.5	2		2.7	
2.2		1.5		1.4		2.9	
1.3		1.2		0.9		4	
1.5		1.1		1.7		3.8	
2.9				4.4		1.4	
1				1		1.7	
1.8				1.9		0	
2.4				1.8		2	
1				1.3			
1.1				1			
1.1				1.7			
1				0.5			
1.4				1			
1.1				4.1			
2.5				1.1			
				1.9			

Supplementary data sheet 3.1

The number of neurons double stainied with TMEM119 and TNFα in CA1

Sham	Injection	WTD
2.3	8	4.8
1.9	7.1	2.9
3.1	7.2	8.4

The number of neurons double stainied with TMEM119 and TNFα in CA3

Sham	Injection	WTD
1.9	19	3.2
2.1	12.4	2.2
1.6	9.6	2.9

Area of the cell body of microglia in CA1

Sham	Injection	WTD
1476	5202	3162
1664	3616	1908
1076	4130	2558
708	4854	2000
1564	6468	3333
1530	3722	5760
970	4458	2436
882	5786	3552
720	4040	3798
1158	4278	5906
474	3708	3886
1446	4992	2042
1402	4038	6050
856	2546	2534
2198	3741	5518
2010	3574	2792
1434	3692	3164
1226	4644	2992
1680	3830	4400
1308	4650	3128
988	5136	2652
1138	6220	5382
924	3944	2262
1268	3670	2434
1050	5112	3264
1370	5232	2014
768	5708	1730
1632	3958	4680
4282	7294	4364
1626	5188	2762
1976	5142	3744
2790	3486	1312
1500	4240	1088
1470	4290	2336
1920	3758	4004
2122	4662	1751
2092	4666	2653
2032	1718	2298
1830	3556	3136
1424	4372	2431
1552	2240	2222
1982	2770	3862
2066	4854	
1470	1088	
1616	2776	
1456	2102	
	2246	
	5052	

Area of the cell body of microglia in CA3

Sham	Injection	WTD
2334	2056	1360
1440	1564	998
1552	2406	1420
2000	2754	1200
2214	1158	1612
1162	2720	1415
2118	3944	2272
1888	3918	1634
1204	1936	1118
1282	4708	1376
1454	3844	1296
1472	3438	816
1070	4232	884
1536	3594	1238
1248	4106	1132
2410	5245	1866
1066	2234	1644
1206	3444	1046
996	7998	1456
1642	4448	1422
1386	5298	686
1204	3408	1898
1276	6385	1566
1322	5820	1464
1438	3400	1242
2152	3196	756
2124	4276	1450
1552	5770	1154
1404	5088	1408
1504	3258	1399
1426	4652	2712
1860	2992	1462
1962	2344	1842
1998	2854	2810
758	4324	1562
1152	4354	2256
806	3986	3360
1226	2946	1052
1044	2280	1628
1322	4476	1678
1160	3700	2268
1268	3584	974
2406	2440	1760
1260	3994	1568
1144	3740	1566
1414	4894	866
944	4148	432
864	5514	1170

3552	1550	5944	1446
2770	1914	7040	2320
	1180	3162	698
	1554	6782	1584
	1134	3690	1724
	2137	2840	1174
	1856	4738	1590
	1324	6046	1640
	2212	4400	2538
	1200	4022	1866
	1206	4186	1048
	1440	6122	1126
	1828	8630	1150
	1228	4938	1878
	1658	4528	1554
	1694	4066	1720
	1094	7442	1134
	2022	8500	1448
	1544	7548	1432
	1889	5296	1436
	1710	4552	1100
	1180	3550	738
	1222	5736	1060
	1344	3918	1720
	1582		
	1542		
	1082		
	1090		
	1734		

Supplementary data sheet 3.2

The Total length of the dendrites of neurons

Groups	Sham			Injection			WTD		
Brain nuclei	Mean	SEM	N	Mean	SEM	N	Mean	SEM	N
CA1-Apical	1508	81.74	34	503.7	22.56	31	1211	61.95	24
CA1-Basal	1279	78.83	30	398	19.87	30	1222	84.06	22
CA3-Apical	845.4	40.43	38	231	19.13	21	849.2	70.56	20
CA3-Basal	984.1	47.8	29	474.9	23.4	34	1030	53.91	24

The intersections of the dendrites of neurons in CA1

Apical side	Sham			SNL			WTD		
	Mean	SEM	N	Mean	SEM	N	Mean	SEM	N
20	1.235294	0.103923	34	1.032	0.03226	31	1.458333	0.134315	24
40	1.676471	0.182675	34	1.065	0.04485	31	1.208333	0.103895	24
60	2.558824	0.283822	34	1.161	0.06715	31	1.291667	0.127393	24
80	3.411765	0.295895	34	1.419	0.1114	31	1.583333	0.179741	24
100	4.029412	0.320108	34	1.645	0.1506	31	2.041667	0.203939	24
120	4.441176	0.330265	34	1.935	0.1534	31	2.458333	0.269051	24
140	4.617647	0.304496	34	2	0.1227	31	2.75	0.264095	24
160	4.882353	0.320885	34	2.172	0.1651	29	3.083333	0.275291	24
180	4.941176	0.24597	34	2.241	0.1766	29	3.333333	0.310835	24
200	4.647059	0.256406	34	2.185	0.2	27	3.375	0.261077	24
220	4.441176	0.319288	34	1.96	0.1869	25	3.333333	0.245737	24
240	4.151515	0.31116	33	1.85	0.1817	20	3.5	0.248182	24
260	3.96875	0.346307	32	1.588	0.15	17	3.416667	0.240144	24
280	3.741935	0.30398	31	1.467	0.1333	15	3.25	0.302466	24
300	3.483871	0.34037	31	1.231	0.1216	13	3.041667	0.303835	24
320	3	0.290957	28	1.273	0.1408	11	2.875	0.235542	24

The intersections of the dendrites of neurons in CA1

Basal side	Sham			SNL			WTD		
	Mean	SEM	N	Mean	SEM	N	Mean	SEM	N
20	2.810811	0.200741	37	2.233	0.1708	30	2.227273	0.196636	22
40	4.459459	0.221226	37	2.733	0.1585	30	3.318182	0.152701	22
60	5.459459	0.283677	37	3.233	0.1413	30	3.954545	0.153984	22
80	6.162162	0.298931	37	3	0.2144	30	4.409091	0.193611	22
100	5.972973	0.338887	37	2.429	0.1493	28	4.5	0.261117	22
120	6.142857	0.330883	35	1.917	0.1583	24	4.772727	0.270736	22
140	5.914286	0.430695	35	1.6	0.1338	20	4.590909	0.299054	22
160	5.40625	0.401257	32	1.467	0.1652	15	4.454545	0.346456	22
180	4.344828	0.382099	29	1.3	0.1528	10	4.272727	0.355427	22

The intersections of the dendrites of neurons in CA3

Apical side	Sham			SNL			WTD		
	Mean	SEM	N	Mean	SEM	N	Mean	SEM	N
20	2.024	0.1924	50	1	0	21	1.88	0.1639	31
40	2.694	0.2449	50	1.238	0.09524	21	2.349	0.1693	31
60	3.656	0.251	50	1.571	0.2135	21	3.368	0.2003	31
80	4.388	0.2606	50	1.762	0.1528	21	4.125	0.2193	31
100	4.726	0.2927	50	1.55	0.1141	20	4.234	0.1901	31
120	4.491	0.3117	49	1.714	0.2206	14	3.901	0.2175	31
140	4.136	0.3049	47	1.5	0.1946	12	3.483	0.2507	31
160	3.198	0.2498	44	1.444	0.2422	9	3.341	0.2467	31
180	2.908	0.2519	34	1.2	0.2	5	2.485	0.2153	31

The intersections of the dendrites of neurons in CA3

Basal side	Sham			SNL			WTD		
	Mean	SEM	N	Mean	SEM	N	Mean	SEM	N

20	2.581	0.1674	38	1.8	0.168	35	2.632	0.1813	28
40	4.303	0.2913	38	2.4	0.1843	35	3.527	0.2497	28
60	5.659	0.2974	38	2.886	0.1913	35	4.284	0.2392	28
80	5.949	0.3578	38	3	0.169	35	4.657	0.2046	28
100	5.372	0.3643	37	2.824	0.1765	34	4.626	0.2053	28
120	4.587	0.3641	35	2.879	0.1834	33	3.707	0.1751	28
140	3.455	0.3559	31	2.258	0.2174	31	3.059	0.2042	28

Supplementary data sheet 3.3

50% paw withdral threshold (g) tested 1hr after gavage

Grouping	Sham			Injection			WTD			PGB		
Day	Mean	SEM	N	Mean	SEM	N	Mean	SEM	N	Mean	SEM	N
D0	0.4072	0.0114	8	0.4169	0.0114	8	0.4169	0.008756	8	0.4072	0.0114	8
D7 (1)	0.412	0.01033	8	0.05224	0	8	0.05083	0.005858	8	0.3221	0.007325	8
D9 (3)	0.4024	0.01211	8	0.05224	0	8	0.1129	0.01039	8	0.2681	0.03053	8
D11 (5)	0.4217	0.01211	8	0.04726	0.001882	8	0.14	0.02605	8	0.189	0.03699	8
D13 (7)	0.412	0.01033	8	0.04726	0.001882	8	0.1982	0.03413	8	0.1572	0.03494	8
D15 (9)	0.4024	0.009662	8	0.04602	0.001822	8	0.3537	0.01723	8	0.1525	0.03697	8

Forced swimming - immobility time (Sec)

Grouping	Sham			Injection			WTD			PGB		
Day	Mean	SEM	N	Mean	SEM	N	Mean	SEM	N	Mean	SEM	N
D0	192.4	2.645	8	192.3	3.358	8	191.6	3.017	8	192.5	2.809	8
D7 (1)	187.3	3.707	8	221.8	1.897	8	224.5	2.338	8	224.5	1.813	8
D15 (9)	198.6	2.618	8	225.9	1.757	8	203.9	2.741	8	227.6	3	8

Tail suspension - immobility time (Sec)

Grouping	Sham			Injection			WTD			PGB		
Day	Mean	SEM	N	Mean	SEM	N	Mean	SEM	N	Mean	SEM	N
D0	77.22	4.189	8	77.78	3.558	8	73.33	3.756	8	78.56	3.375	8
D7 (1)	67.5	5.089	8	134.6	4.255	8	138	6.31	8	135.4	5.919	8
D15 (9)	78.13	5.74	8	141.9	8.225	8	87.13	4.875	8	139.6	8.836	8

The open field - central duration time (Sec)

Grouping	Sham			Injection			WTD			PGB		
Day	Mean	SEM	N	Mean	SEM	N	Mean	SEM	N	Mean	SEM	N
D0	21.57	1.289	8	22.27	1.167	8	22.95	1.54	8	23.19	1.149	8
D7 (1)	19.93	1.587	8	5.259	0.5816	8	4.748	0.6096	8	3.803	0.4742	8
D15 (9)	23.42	0.9383	8	3.896	0.6475	8	20.46	1.085	8	8.938	0.8488	8

Supplementary data sheet 4.1

The number of neurons double stainied with TMEM119 and TNFα in CA1

Sham	SNL	WTD	WTD-TNFα
1.7	9.9	3.3	6.9
2	7.4	2.9	7.1
2.3	11.2	3.7	8.7

The number of neurons double stainied with TMEM119 and TNFα in CA3

Sham	SNL	WTD	WTD-TNFα
1.3	17.1	1.2	15.1
1.9	12	2.1	15.2
1.7	9.9	2.3	12.1

Area of the cell body of microglia in CA1

Sham	Injection	WTD	WTD-TNFα
1632	4080	3692	2824
4282	4830	3370	3426
1626	6466	2692	3636
1976	4680	3224	3834
2790	2856	1264	3414
1500	4404	1880	3722
1470	4042	2002	3548
1920	4550	1436	4168
2122	2924	2140	3384
2092	4416	2962	2886
2032	5184	2004	3122
1830	3742	2202	2604
1424	4140	1972	4418
1552	5146	3468	4894
1982	4794	1900	3382
2066	3180	2993	3386
1470	3946	3260	5762
1616	5128	4720	6076
1456	5056	2558	4075
1102	7406	5574	3985
1484	6236	3430	2962
1300	3618	5300	4095
2038	7206	3806	5639
1573	4122	2822	4405
1472	4038	3112	3682
1684	6203	3000	5835
1900	3928	3750	4989
1804	3810	1542	3086
1604	4801	2412	2804
1271	6783	1846	3279
1100	5260	3322	6377
1765	4965	2118	4380
1205	6654	2646	5063

Area of the cell body of microglia in CA3

Sham	Injection	WTD	WTD-TNFα
2334	3346	1394	5458
1440	2472	2068	3984
1552	2873	1244	2882
2000	3338	1044	3144
2214	5208	956	2418
1162	5148	1648	5977
2118	6746	1492	4532
1888	2436	842	6138
1204	5310	1248	6305
1282	5092	1302	5418
1454	4480	976	6534
1472	3676	1592	5662
1070	4460	1424	3828
1536	4844	1042	5060
1248	4954	1610	2615
2410	3600	1080	3532
1066	6330	1624	3580
1206	7458	1058	5844
996	4362	1600	5268
1642	4056	1414	3788
1386	4414	986	3596
1204	4030	1556	5508
1276	3568	1112	4054
1322	3682	1044	2312
1438	3901	1634	6018
2152	7372	1316	5816
2124	3466	1226	3352
1552	2970	1564	3100
1404	3889	3659	3946
1504	4160	1334	4122
1426	4889	1828	6224
1860	5136	1910	4728
1962	5000	1570	4042
1998	5806	2016	3798
758	5862	1848	3632
1152	4164	1406	4196
806	5276	1744	5060
1226	5744	2363	4766
1044	5446	2103	3344
1322	4000	1610	4938
1160	3210	1973	5766
1268	4190	1483	7204
2406	5970	1238	5496
1260	7044	1326	5418
1144	4110	2352	3454
1414	4586	1122	3598
944	4834	1620	3804
864	4578	1262	5558

1550	3974	1664	5476
1914	4320	2094	5628
1180	4756	1824	4432
1554	4970	1864	3570
1134	5478	1474	3836
2137	7266	2068	5664
1856	6040	1798	3452
1324	3860	1542	4279
2212	3542	1436	4770
1200	4312	1540	3414
1206	4728	1824	7108
1440	3516	1106	3922
1582	4388	1576	5470
1542	5456	1050	5334
1082	5932	2076	3378
1090	4442	1436	3520
1734	5603	1172	8740
2022	5416	1938	6653
1544	4210	1704	4070
1889	4880	1152	5666
1710	2896	2008	5532
1180	5238	1384	5604
1222	3934	934	6758
1344	5066	1550	5880

Supplementary data sheet 4.2

The Total length of the dendrites of neurons

	Sham			Injection			WTD			WTD-TNFα		
	Mean	SEM	N	Mean	SEM	N	Mean	SEM	N	Mean	SEM	N
**CA1-Apical**	1508	81.74	34	663.5	23.78	51	1083	70.66	26	664.1	39.73	41
**CA1-Basal**	1279	78.83	30	722.9	27.06	62	1102	67.28	22	644.4	39.49	38
**CA3-Apical**	845.4	40.43	38	309.2	16.94	43	822	41.65	42	355.8	26.37	39
**CA3-Basal**	917.6	41.66	28	513.7	30.19	35	816.9	39.89	37	474.5	27.71	52

The intersections of the dendrites of neurons in CA1

Apical side	Sham			SNL			WTD			WTD-TNFα		
	Mean	SEM	N	Mean	SEM	N	Mean	SEM	N	Mean	SEM	N
20	1.2353	0.103923	34	1.145	0.044737	76	1.16	0.0945	25	1.2439	0.090933	41
40	1.6765	0.182675	34	1.539	0.10993	76	1.36	0.14	25	1.4878	0.135799	41
60	2.5588	0.283822	34	1.776	0.139672	76	1.88	0.1943	25	2.1463	0.176556	41
80	3.4118	0.295895	34	2.092	0.169807	76	2.52	0.259	25	2.6341	0.199866	41
100	4.0294	0.320108	34	2.382	0.198209	76	3.08	0.2996	25	3.0244	0.205081	41
120	4.4412	0.330265	34	2.553	0.195919	76	3.52	0.3323	25	3.122	0.237351	41
140	4.6176	0.304496	34	2.697	0.195583	76	4.12	0.3972	25	2.9024	0.220324	41
160	4.8824	0.320885	34	2.645	0.194399	76	3.96	0.3807	25	2.8718	0.217889	39
180	4.9412	0.24597	34	2.711	0.201587	76	4.32	0.3684	25	2.5833	0.215933	36
200	4.6471	0.256406	34	2.566	0.176054	76	3.88	0.3844	25	2.2121	0.21654	33
220	4.4412	0.319288	34	2.395	0.164454	76	3.52	0.3372	25	2.08	0.230362	25
240	4.1515	0.31116	33	2.4	0.151865	75	3.44	0.327	25	2.375	0.256174	16
260	3.9688	0.346307	32	2.27	0.112685	74	3.04	0.2971	25	2.5625	0.257694	16
280	3.7419	0.30398	31	2.271	0.125309	70	2.8	0.3109	25	2	0.239046	15
300	3.4839	0.34037	31	2.045	0.087524	66	2.5833	0.3292	24	1.9	0.276887	10
320	3	0.290957	28	1.879	0.095506	58	2.381	0.3483	21	2.1429	0.26082	7

The intersections of the dendrites of neurons in CA1

Basal side	Sham			SNL			WTD			WTD-TNFα		
	Mean	SEM	N	Mean	SEM	N	Mean	SEM	N	Mean	SEM	N
20	2.8108	0.200741	37	2.391	0.119143	64	2.6364	0.2422	22	2.5789	0.158368	38
40	4.4595	0.221226	37	3.531	0.129806	64	4.1818	0.2244	22	3.7105	0.155084	38
60	5.4595	0.283677	37	3.906	0.143854	64	5.1818	0.3521	22	4.1053	0.195211	38
80	6.1622	0.298931	37	4.141	0.150836	64	5.7273	0.3302	22	4.2105	0.200135	38
100	5.973	0.338887	37	4.188	0.164984	64	5.8636	0.3561	22	4.1579	0.237119	38
120	6.1429	0.330883	35	4.127	0.17888	63	5.9545	0.3255	22	4.1471	0.246662	34
140	5.9143	0.430695	35	3.661	0.186741	62	5.8636	0.3739	22	3.4545	0.301797	33
160	5.4063	0.401257	32	3.07	0.198458	57	5.0455	0.4437	22	3	0.350823	26
180	4.3448	0.382099	29	2.28	0.189608	50	4.0455	0.4534	22	2.1739	0.318421	23
200	3.3214	0.40517	28	1.737	0.154055	38	3.1176	0.4917	17	1.4545	0.207305	11
220	2.6667	0.374295	24	1.381	0.12866	21	2.25	0.3917	12	1	0	2

The intersections of the dendrites of neurons in CA3

Apical side	Sham			SNL			WTD			WTD-TNFα		
	Mean	SEM	N	Mean	SEM	N	Mean	SEM	N	Mean	SEM	N
20	2.024	0.1924	50	1.511	0.1217	43	1.692	0.1347	42	1.4872	0.10942	39
40	2.694	0.2449	50	2.051	0.1372	43	2.435	0.2436	42	1.9231	0.157514	39
60	3.656	0.251	50	2.303	0.1584	42	3.537	0.2672	42	2.2632	0.134324	38
80	4.388	0.2606	50	2.224	0.1721	41	4.283	0.3031	42	2.3421	0.16559	38
100	4.726	0.2927	50	2.151	0.1639	33	4.539	0.3041	42	2.3939	0.230034	33
120	4.491	0.3117	49	1.994	0.1651	29	4.559	0.2855	42	2.0345	0.240498	29
140	4.136	0.3049	47	1.792	0.1664	23	4.098	0.293	42	2.1304	0.276228	23
160	3.198	0.2498	44	1.315	0.1508	16	3.399	0.2551	40	2.1765	0.260589	17
180	2.908	0.2519	34	1.297	0.1521	9	2.553	0.2433	34	2.0909	0.284591	11

The intersections of the dendrites of neurons in CA3

Basal side	Sham			SNL			WTD			WTD-TNFα		
	**Mean**	**SEM**	**N**	**Mean**	**SEM**	**N**	**Mean**	**SEM**	**N**	**Mean**	**SEM**	**N**
20	2.581	0.1674	38	1.884	0.1062	43	3.293	0.2424	41	2.1923	0.116635	52
40	4.303	0.2913	38	2.213	0.135	42	5.121	0.2954	41	2.8846	0.144413	52
60	5.659	0.2974	38	2.454	0.1883	35	6.313	0.2839	41	3.1923	0.164859	52
80	5.949	0.3578	38	2.633	0.2178	27	6.746	0.3814	41	3.28	0.189608	50
100	5.372	0.3643	37	2.67	0.2263	23	5.864	0.428	40	3.0638	0.183512	47
120	4.587	0.3641	35	2.134	0.2948	19	4.697	0.4609	38	2.8571	0.203259	42
140	3.455	0.3559	31	1.961	0.3009	12	3.562	0.4552	34	2.6176	0.215342	34

Supplementary data sheet 4.3

50% paw withdral threshold (g) tested 1hr after gavage

Group	Sham			SNL			WTD			WTD-TNFα		
Day	Mean	SEM	N	Mean	SEM	N	Mean	SEM	N	Mean	SEM	N
0	0.4775	0.007846	8	0.4667	0.007846	8	0.4828	0.007018	8	0.4721	0.008103	8
1	0.42652	0.00707	8	0.16748	0.010237	8	0.1827	0.008958	8	0.155721	0.015326	8
4	0.4469	0.011984	8	0.03796	0.006888	8	0.07051	0.016974	8	0.088071	0.012287	8
7	0.42652	0.00707	8	0.03626	0.002184	8	0.03874	0.015343	8	0.041497	0.002684	8
9	0.44101	0.006324	8	0.02515	0.00387	8	0.17487	0.03053	8	0.142052	0.026405	8
12	0.441	0.006324	8	0.02398	0.002525	8	0.2413	0.02777	8	0.08813	0.01957	8
15	0.441	0.006324	8	0.03987	0.002135	8	0.3057	0.00605	8	0.09146	0.01237	8
18	0.4415	0.01005	8	0.02369	0.00177	8	0.3255	0.01244	8	0.05033	0.001249	8
21	0.4313	0.007302	8	0.02922	0.003554	8	0.3534	0.04428	8	0.05033	0.001249	8

Forced swimming - immobility time (Sec)

Group	Sham			SNL			WTD			WTD-TNFα		
Day	Mean	SEM	N	Mean	SEM	N	Mean	SEM	N	Mean	SEM	N
D0	191.2	2.602	10	190.2	3.589	10	191.7	2.661	10	190.3	3.575	10
D7	196.1	3.413	10	225.7	1.563	10	195.8	3.651	10	193	3.308	10
D21	192.4	2.604	10	225.7	1.62	10	197.4	3.074	10	226.5	1.905	10

Tail suspension - immobility time (Sec)

Group	Sham			SNL			WTD			WTD-TNFα		
Day	Mean	SEM	N	Mean	SEM	N	Mean	SEM	N	Mean	SEM	N
D0	75.8	4.855	10	79.1	3.371	10	78.2	3.823	10	80.1	3.743	10
D7	86.7	3.307	10	132.8	9.871	10	85.5	4.235	10	86.6	4.009	10
D21	52.1	6.439	10	177.3	6.596	10	72.8	10.81	10	110.2	7.517	10

The open field - central duration time (Sec)

Group	Sham			SNL			WTD			WTD-TNFα		
Day	Mean	SEM	N	Mean	SEM	N	Mean	SEM	N	Mean	SEM	N
D0	24.59	1.803	10	21.95	1.016	10	22.73	1.504	10	24.41	1.973	10
D7	23.85	1.65	10	7.163	0.569	10	23.62	1.939	10	23.43	1.856	10
D21	22.06	1.16	10	9.929	0.9658	10	20.66	2.213	10	6.626	0.7882	10

Supplementary data sheet 5

AMPAR1/ GAD65 ratio in CA1

	**Sham**	**SNL**	**SNL-WTD**	**WTD-TNFα**	**Injection**	**Injection-WTD**
1	25	0.9	21.6	2.9	1.2	8.7
2	17.4	1.8	9.7	3.1	2.1	16.7
3	23.8	1.2	20	1.9	2	12.5

AMPAR1/ GAD65 ratio in CA3

	**Sham**	**SNL**	**SNL-WTD**	**WTD-TNFα**	**Injection**	**Injection-WTD**
1	5.2	0.7	3.9	0.4	0.5	5.9
2	4	1.3	4.5	0.8	0.5	4.5
3	5.3	2.1	5.8	1	0.8	5.5

AMPAR2/ GAD65 ratio in CA1

	**Sham**	**SNL**	**SNL-WTD**	**WTD-TNFα**	**Injection**	**Injection-WTD**
1	17	0.7	10	2.5	1.1	9.5
2	30.1	1.3	11.3	2.9	1.9	10.9
3	19.5	1.1	17.1	1.9	2.2	13.1

AMPAR2/ GAD65 ratio in CA3

	**Sham**	**SNL**	**SNL-WTD**	**WTD-TNFα**	**Injection**	**Injection-WTD**
1	5	0.7	2.8	0.5	0.4	4.2
2	5.3	0.9	3.6	0.8	0.6	4.8
3	4.7	1.4	5.6	1	0.7	5
